# Multifunctional pectin derivatives as anticancer agents in colorectal cancer via synthesis, computational insights, and modulation of NRF2/HO-1, HIF-1α, and VEGF/PDGF-D signaling pathways

**DOI:** 10.1038/s41598-025-32107-6

**Published:** 2026-02-13

**Authors:** Ghada H. Elsayed, Asmaa M. Fahim

**Affiliations:** 1 Hormones Department, Medical Research and Clinical Studies Institute, National Research Centre, Dokki, P.O. Box.12622, Cairo, Egypt; 2 Stem Cell Lab, Centre of Excellence for Advanced Sciences, National Research Centre, Dokki, P.O. Box.12622, Cairo, Egypt; 3 Department of Green Chemistry, National Research Centre, Dokki, P.O. Box.12622, Cairo, Egypt

**Keywords:** Pectin derivatives, Colorectal cancer, Cytotoxicity, Oxidative stress, Angiogenesis, Theoretical studies, Biochemistry, Cancer, Chemical biology, Chemistry, Drug discovery

## Abstract

This research describes the design, synthesis, characterization, and biological assessment of new Pectin-based Hydrazide and Oxadiazole derivatives as possible anticancer agents. The chemical modification of native Pectin was accomplished using a sequence of esterification, Hydrazide formation, and cyclization with carbon disulfide to yield Pectin Hydrazide (3) and Pectin Oxadiazole (5), confirmed using FT-IR, ^1^H/^13^C NMR, elemental, SEM, and TGA analyses. The in vitro anticancer activity of the synthesized Pectin derivatives was evaluated against HepG2 and Caco2 cancer cells using the neutral red uptake test. Pectin Oxadiazole (IC_50_ = 23.5 μg/mL) and Pectin Hydrazide (IC_50_ = 39.5 μg/mL) demonstrated the most potent cytotoxic effects against Caco2 cells after 48 h. ELISA assays showed significant reductions in ROS activity and HO-1 protein levels, while qRT-PCR confirmed significant suppression of NRF2, HIF-1α, VEGF, and PDGF-D gene expression in Caco2 cells treated with these Pectin derivatives. Molecular docking and 100-ns molecular dynamics simulations against several cancer-related targets (PDB IDs: 1lc8, 1n3u, 1u6d, 3nl0, 5k5x) demonstrated that Pectin Oxadiazole had a stronger binding affinity with greater structure stability and greater ΔG__bind_ (− 34 to − 82 kcal/mol) along with a much lower RMSD profile. Additionally, density functional theory (DFT/B3LYP/6-31G) calculations suggested that the presence of N- and S-containing heterocycles resulted in lower HOMO–LUMO gaps and improved dipole moments, thereby increasing electronic reactivity and charge transfer characteristics. Taken together, the Pectin Oxadiazole and Pectin Hydrazide derivatives show combined cytotoxic, antioxidant, and anti-angiogenic activities, thus warranting further investigation toward novel multifunctional agents for the treatment of colorectal cancer.

**Supplementary Information:**

The online version contains supplementary material available at 10.1038/s41598-025-32107-6.

## Introduction

Pectin extracted from a variety of plant sources and modified by heat and/or pH has demonstrated significant anticancer activity^1^. Several in vitro studies reported that Pectin inhibits the proliferation of melanoma and prostate cancer cells, while in vivo evidence showed that oral administration of soluble Pectin fragments suppressed the growth and metastasis of transplanted colon tumors in mice. These findings suggest that Pectin and its derivatives may represent promising dietary agents for cancer prevention and therapy^2,3^. Globally, cancer remains the second leading cause of mortality, with approximately 19 million new cases and 10 million deaths recorded in 2020^4,5^. Both colorectal cancer (CRC) and hepatocellular carcinoma (HCC) are among the most prevalent gastrointestinal cancers, characterized by uncontrolled cell proliferation, resistance to apoptosis, and enhanced angiogenesis. A common pathogenic hallmark is oxidative stress, which arises from excessive generation of reactive oxygen species (ROS) and reactive nitrogen species (RNS)^6,7^. At high levels, ROS induce DNA damage and genomic instability, whereas at moderate levels they act as signaling mediators that sustain tumor growth. The nuclear factor erythroid 2-related factor 2 (NRF2) plays a dual role in cancer: while it protects normal cells from oxidative injury, sustained activation in tumors promotes proliferation, therapeutic resistance, and metastatic invasion. One of its key downstream targets, heme oxygenase-1 (HO-1), functions as a phase II detoxification enzyme, providing antioxidant defense by degrading pro-oxidant heme. However, the NRF2/HO-1 axis has been shown to support tumor survival and angiogenesis at advanced stages, reflecting its “double-edged sword” role in cancer biology^8,9^. Vascular endothelial growth factor (VEGF), secreted by malignant colon cells, drives angiogenesis, thereby supporting rapid tumor growth and metastasis^10, 11^. Elevated VEGF levels have been reported in AOM-induced colon cancer, correlating with increased neovascularization and aberrant crypt foci (ACF) formation. Importantly, inhibition of the NRF2 pathway suppresses hypoxia inducible factor (HIF-1α) accumulation, leading to downregulation of VEGF and its target genes, ultimately reducing tumor angiogenesis and growth under hypoxic conditions in mice^13–15^. Similarly, platelet-derived growth factor-D (PDGF-D) is a relatively new member of the PDGF family and has emerged as a central mediator of angiogenesis, cell proliferation, migration, tissue repair, regeneration, and fibrosis. PDGF-D over expression has been linked to tumor progression, angiogenesis, and stromal activation**,** with deregulated PDGF-D signaling contributing to enhanced tumor growth and metastatic potential. Furthermore, studies reveal that PDGF-D expression correlates with the prognosis of different tumors^16^.

This study focuses on the design, synthesis, and characterization of new derivatives of Pectin-based Hydrazide and Oxadiazole, and their anticancer activities on hepatocellular (HepG2) and colorectal (Caco2) cancer cell lines. It also aims to elucidate their mechanisms of action through the modulation of oxidative stress markers (ROS and HO-1) and the mRNA expression of important angiogenesis-related genes (NRF2, HIF-1α, VEGF, and PDGF-D). In addition, we performed complementary molecular docking, molecular dynamics simulations, and DFT studies to correlate our experimental cytotoxicity with the structural, electronic, and binding features of the derivatives in order to establish a structure–activity relationship that would assist in identifying the most promising derivatives for further testing of colorectal cancer therapy.

## Experimental section

### Instruments and techniques

The Gallenkamp melting point apparatus was used for measuring melting points. Moreover, Shimadzu FT-IR 8101 PC infrared spectrophotometer recorded the IR spectra. The ^1^H NMR and ^13^C NMR spectra were determined in DMSO-D₆ at 300 MHz on a Varian Mercury VX 300 NMR spectrometer (^1^H at 300 MHz, ^13^C at 75 MHz) using trimethylsilane as an internal standard. Scanning electron microscopes (SEM) were investigated using a JEOL JXA-840A electron probe microanalyzer. Samples were air-dried before imaging, and images were obtained at an accelerating voltage of 10–15 kV. Thermogravimetric analysis (TGA) was performed to assess thermal stability and degradation properties of native pectin and its synthesized derivatives (Pectin Methyl Ester, Pectin Hydrazide, and Pectin Oxadiazole). The measurements were performed on a Shimadzu DTG-60H thermogravimetric analyzer (Japan). Samples approximately 5–10 mg in mass were prepared from each sample as a finely powdered sample and placed into a platinum crucible, and heated under a nitrogen atmosphere (50 mL min^−1^) from 30 to 600 °C at a 10 °C min^−1^ heating rate.

### Chemicals and reagents

Standard suppliers provided the Pectin (analytical grade), methanol, concentrated sulfuric acid, hydrazine hydrate (N_2_H_4_·H_2_O, 80%), carbon disulfide, potassium hydroxide, and hydrochloric acid, and were used without further purification. Solvents were purified, if necessary, before use. All reactions were performed in an open reflux with magnetic stirring.

### Synthesis of Pectin Ester (2)

Pectin (1) (10 g) was suspended in methanol (100 mL), and then a few drops of concentrated H_2_SO_4_ were added as a catalyst. The mixture was refluxed for 6–8 h under continuous stirring. Following completion, the mixture was cooled to room temperature, neutralized with sodium bicarbonate (NaHCO_3_), and then poured into excess cold ethanol. The precipitate was filtered, washed, and dried under vacuum to yield Pectin Methyl Ester (2): methyl (2S,4R,5R,6R)-6-(((2R,3R,5S,6R)-4,5-dihydroxy-2-(methoxycarbonyl)-6-methyltetrahydro-2H-pyran-3-yl)oxy)-4,5-dihydroxy-3-methoxytetrahydro-2H-pyran-2-carboxylate(2), white color, yield = 77%, m.p = 213–215 °C, FT-IR (KBr): ν, 3465(OH), 2893(CH), 1763(C=O), 1688(C=O) cm^−1^, 1H NMR (400 MHz, DMSO-d_6_) δ: 5.02 (m, 1H, CH), 4.32 (m, 2H, CH_2_), 4.14 (m, 2H, CH_2_), 3.55 (m, 2H, CH_2_, glucose), 3.22 (s, 3H, OCH_3_),^13^C NMR (100 MHz, DMSO-d_6_) δ: 166.0 (C=O), 108.0 (CH), 77.0 (CH_2_), 66.7 (CH_2_), 55.0 (OCH_3_), 17.0 (CH_3_).

### Preparation of Pectin Hydrazide (3)

Pectin Methyl Ester (1.0 g) was dispersed in ethanol (30 mL) and stirred at room temperature for 20 min. Hydrazine hydrate (80%, 3 mL) was added dropwise, and the reaction mixture was heated under reflux at 70 °C for 6 h. Afterwards, the mixture was allowed to cool and was subsequently poured into excess cold acetone to yield the product, which was collected by filtration, washed several times with acetone, and dried in a vacuum oven at 40 °C to give (2R,3R,5S,6R)-3-(((2R,3R,4R,6S)-6-(hydrazinecarbonyl)-3,4-dihydroxy-5-methoxytetrahydro-2H-pyran-2-yl)oxy)-4,5-dihydroxy-6-methyltetrahydro-2H-pyran-2-carbohydrazide, Pectin Hydrazide (3), Pale yellow color, yield = 63%, m.p = 250–252 °C, FT-IR (KBr): ν, 3465(OH), 3300(NH), 3190(NH), 2893(CH), 1672(C=O), 1590(amide II, coupled N–H bending and C-N stretching of the hydrazide group cm^−1^, ^1^H-NMR (DMSO): δ, 9.49–9.39 (s, NH, D_2_O exchangeable), 5.70–5.30 (m, H-1, CH), 5.30–5.05 (m, H-2/H-3, CH), 4.40–3.90 (m, NH, exchangeable), 3.80–3.10 (m, CH₂/CH, glycose), ^13^CNMR(DMSO): δ, 166.1–166.2 (C=O), 108.0 (C-1), 80.1–74.4 (C-2, C-3, C-4), 68.7–67.4 (C-5), 49.4 (CH₂-NH),

### Synthesis of the thioyl salt of Hydrazide

Pectin Hydrazide (3) (2 g) was weighed and suspended in ethanol (40 mL). CS₂ (5 mL) was added dropwise in the presence of KOH (2 g, in 20 mL of ethanol). The mixture was refluxed for 12 h and then cooled. The potassium salt of Pectin Hydrazide (4) was filtered, washed, and then dried.

### Synthesis of Pectin Oxadiazole (5)

A suspension of Pectin Hydrazide (4) salt (2 g) was prepared in ethanol (50 mL), and concentrated hydrochloric acid (HCl, 5 mL) was added. The mixture was refluxed for 6 h, cooled and the precipitate was then collected by filtration and successively washed with ethanol and water, and dried under vacuum to give (2R,3R,4R,6S)-2-(((2R,3R,5S,6R)-4,5-dihydroxy-2-(5-mercapto-1,3,4-oxadiazol-2-yl)-6-methyltetrahydro-2H-pyran-3-yl)oxy)-6-(5-mercapto-1,3,4-oxadiazol-2-yl)-5-methoxytetrahydro-2H-pyran-3,4-diol(5): Pectin Oxadiazole (5), yellow color, yield = 66%, m.p = 270–272 °C, FT-IR (KBr): ν = 3239(OH), 2832(CH), 1623–1590(C=N) stretching,1050–1150 (C–O–C/C–O–N, glycosidic), 690 cm^−1^ (C=S stretching, thione form), ^1^H-NMR (DMSO): δ, 12.75 (s, 1H, SH, D_2_O,exchangable), 5.93–5.34 (m, glucose CH), 4.40–3.90 (m, CH-O and CH_2_-O), 3.50–3.10 (m, CH/CH₂ glucose), ^13^CNMR(DMSO): δ 158.9, 158.8 (C=S/C=N) 127.0, 115.8, 114.8, 113.2 (CH oxadiazole ring), 88.6, 86.4, 79.6, 72.2, 68.5, 68.3 (CH_2_ Pectin).

### Biological evaluation

#### Cell culture and growth conditions

HepG2 (human hepatocellular carcinoma) and Caco2 (human colorectal cancer) cell lines were acquired from the American Type Culture Collection (ATCC). HepG2 cells were cultivated in DMEM, whereas Caco2 cells were grown in RPMI-1640 media. Both cultures were supplemented with 10% fetal bovine serum (FBS), 100 U/mL penicillin, and 100 μg/mL streptomycin sulfate. The cells were maintained at 37 °C in a humidified atmosphere containing 5% CO_2_. Subculturing was done with 0.25% trypsin-0.02% EDTA with about 80% confluence. Cells in the logarithmic growth phase were used in all experiments, and the mean ± SEM of three separate experiments was used to express the results.

#### Neutral red uptake cytotoxicity assay

The neutral red uptake test was used to measure cytotoxicity^17,18^. Caco2 and HepG2 cells were seeded at a density of 1 × 10^4^ cells/well in 96-well plates and given 24 h to adhere. At different doses of 12.5, 25, 50, and 100 µg/mL, the medium was subsequently replaced with a medium containing the test Pectin derivatives: Pectin Hydrazide and Pectin Oxadiazole. Doxorubicin (Dox, MW = 543.5) treated cells at equivalent concentrations were used as positive controls, while untreated cells were used as negative controls. The media was taken out, and the cells were rinsed with DPBS following a 48-h incubation period. After adding 100 µL of a 0.4% neutral red dye solution (in culture media) to each well, the plates were incubated at 37 °C for two hours. After removing the extracellular dye from the cells using DPBS, a destain solution (50% ethanol, 49% water, 1% acetic acid) was added to extract the intracellular neutral red. Using a microplate reader, the absorbance of the extracted dye, which is correlated with the number of live cells, was measured at 450 and 630 nm excitation and emission wavelengths. The correlation between the utilized concentrations and the neutral red intensity value was used to obtain the IC_50_ values.

The percentage of cell growth was calculated as follows in relation to the control:

$$\% \, Cell \, growth \, = \left( {compound \, absorbance/control \, absorbance} \right) \, \times \, 100.$$

#### ROS enzyme activity

In short, 1 × 10^4^ Caco2 cells per well were cultivated in 96-well plates. The IC_50_ values of the Pectin derivatives under investigation were added to the media on the second day. The Human Reactive Oxygen Species (ROS) ELISA Kit (Cat No.: MBS2802062) (MyBioSource, USA) was used to measure the ROS enzyme activity in the cell supernatant, which was expressed as ng/mg protein. Using a microplate reader tuned at 450 nm, optical density was determined.

#### HO-1 protein expression

The level of HO-1 (Heme Oxygenase-1) in Caco2 cells treated with the investigated Pectin derivatives was determined using a Human HO-1 ELISA Kit (Cat No.: E-EL-H2172) (Elabscience, USA), according to the manufacturer’s instructions. Results were expressed as ng/mg protein. The optical density (OD) of each well was measured at 450 nm using a microplate reader.

#### Assessment of gene expression by quantitative real-time PCR (qRT-PCR)

Using qRT-PCR, the impact of the examined Pectin derivatives on gene expression was investigated. Pectin Hydrazide and Pectin Oxadiazole were administered to Caco2 cells (~ 3 × 10^4^ cells/well) at their respective IC_50_ values for 48 h. As directed by the manufacturer, total RNA was extracted using an RNeasy Mini Kit (Qiagen, Germany; Cat. No.: 74104). Using a NanoDrop One UV–Vis spectrophotometer (Thermo Fisher Scientific, USA), the yield and purity of RNA (A_260_/A_280_) were measured. The RevertAid First Strand cDNA Synthesis Kit (Thermo Fisher Scientific, USA; Cat. No.: K1621) was used to create cDNA from 1 µg of total RNA per sample in accordance with the kit’s instructions. A DTlite 4S1 real-time PCR thermocycler (DNA-Technology, Russia) was used to perform quantitative RT-PCR using Maxima SYBR Green qPCR Master Mix (2X) (Thermo Fisher Scientific, USA; Cat. No.: K0221). Table 1 lists the specific primer sequences for NRF2, HIF-1α, VEGF, PDGF-D, and β-actin. The qPCR protocol included 40 cycles of 95 °C for 15 s, 55 °C for 30 s, and 72 °C for 30 s after initial denaturation at 95 °C for 10 min. By normalizing the expression of each target gene to β-actin and comparing treated samples to the control sample (untreated cells) set to 1, the 2^−ΔΔCT^ technique^19^ was used to compute relative gene expression.

**Table 1:** Primer sequences for qRT-PCR.

Gene	Primer forward (5ʹ–3ʹ)	Primer reverse (5ʹ–3ʹ)	References
**β-actin**	CCTTCCTGGGCATGGAGTCCT	GGAGCAATGATCTTGATCTTC	^20^
**NRF2**	TCTCCACAGAAGACCCCAAC	TGCTTTCAGGGTGGTTTTGG	^21^
**HIF-1α**	GCAAGCCCTGAAAGCG	GGCTGTCCGACTTTGA	^20^
**VEGF**	TACCTCCACCATGCCAAGTG	ATGATTCTGCCCTCCTCCTTC	^20^
**PDGF-D**	GTGGAGGAAATTGTGGCTGT	CGTTCATGGTGATCCAACTG	^22^

#### Statistical analysis of data

All data are presented as mean ± SEM from at least three independent experiments. Statistical analyses were performed using Sigma Plot version 11 and GraphPad Prism version 8. Differences between treated cells were evaluated using one-way analysis of variance (ANOVA) followed by Dunnett’s multiple comparisons test. p < 0.05 was considered statistically significant.

### Docking analysis

Molecular docking studies were conducted for the newly developed heterocyclic. We used standard bond lengths and angles within the MOE program, and the analyses were carried out using Discovery Studio Client (version 4.2)^23,24^. After optimizing the geometry, we thoroughly explored various conformations systematically until reaching an RMS gradient of 0.01 Å. The resulting conformations underwent energy minimization using the Confirmation Examination module within Auto Dock Vina. This process was based on the crystal Structure of l-Threonine-O-3-phosphate Decarboxylase from S. enterica complexed with its reaction intermediate (PDB ID:1LC8) (Cancer cell metabolism growth)^25^, Crystal structure of human heme oxygenase 1 (HO-1) in complex with its substrate heme, crystal form B(PDB ID:1N3U)(Oxidative stress, tumor survival, chemoresistance)^26^, Crystal structure of the Kelch domain of human Keap1(PDB ID:1U6D)((NRF2/Keap1 redox regulation))^27^, Crystal structure of human PDGFRA(PDB ID:5K5X)(Angiogenesis and tumor proliferation)^28^, Mutant P44S M296I of Foot-and-mouth disease Virus RNA-dependent RNA polymerase (PDB ID:3NL0)(Structural stability, nucleic acid interaction, general cytotoxicity model)^29^. We conducted nine separate docking simulations using default parameters, and the conformations were selected based on the alignment with overall statistics, E conformation, and compatibility with the pertinent amino acids in the respective protein’s binding pocket^30,31^.

### Molecular dynamics (MD) simulations

To further verify the docking results and evaluate the motion of ligand protein complexes, 100-ns MD simulations were conducted using GROMACS 2023.1 with the CHARMM36 all-atom force field. The CGenFF server was used to generate the ligand topologies. The systems were then placed in a cubic box filled with water (TIP3P) and neutralized with counter-ions (Na⁺/Cl⁻). After energy minimization (using the steepest-descent algorithm), the systems were equilibrated under NVT (300 K) and NPT (1 bar) for 100 ps each. The production MD run was performed for 100 ns with a 2 fs timestep, with applied LINCS constraints and Particle Mesh Ewald (PME) electrostatics, which had a cutoff of 10 Å. After completion of the simulations, the trajectory analyses were for the following: Root Mean Square Deviation (RMSD), Root Mean Square Fluctuation (RMSF), Radius of Gyration (Rg), Solvent Accessible Surface Area (SASA), center-of-mass (COM) distance, and hydrogen bond occupancy. The MM/PBSA binding free energy (ΔG__bind_) was calculated with the g_mmpbsa tool to evaluate the thermodynamic stability of the complexes. VMD 1.9.4, PyMOL, and Grace plotting tools allowed for visualization and submission of trajectories^32,33^.

### Computational analysis

To explore the electronic and structural characteristics of the optimized Pectin derivatives, quantum chemical calculations were performed utilizing Gaussian 09^34^. The optimized geometries and the frequency calculations were calculated at the B3LYP/6-31G(d,p) level of calculation^35^. The FMO, (HOMO) and (LUMO)—were used to calculate the energy gaps (ΔE = E_LUMO_ – E_HOMO_), chemical hardness (η), softness (σ), electronegativity (χ), chemical potential (Π), electrophilicity index (ω), and maximum charge transfer (ΔN_max) according the following equations

1 $$\Delta E = \left( {E_{LUMO} - E_{HOMO} } \right)$$

2 $$\chi = \frac{{ - \left( {E_{HOMO} - E_{LUMO} } \right)}}{2}$$

3 $$\eta = \frac{{\left( {E_{LUMO} - E_{HOMO} } \right)}}{2}$$

4 $$\sigma = 1/\eta$$

5

6 $$S \, = 1/2\eta$$

7 $$\omega = \, Pi^{2} /2$$

8 $$\Delta N \, max = - \, Pi/\eta$$

Two visualization programs, Multiwfn 3.8 and VMD, were used to plot maps of Molecular Electrostatic Potential (MEP), Localized Orbital Locator (LOL), Electron Localization Function (ELF), Non-Covalent Interaction (NCI) isosurface, and Fukui function (f^+^/f^−^). The maps have allowed us to spatially locate possible reactive sites and regions of non-covalent interactions. These properties were also used to relate the computational descriptors to experimental cytotoxicity and docking data to discuss structure–activity relationships and the electronic properties^36^.

## Results and discussion

### Chemistry section

**Scheme 1:**
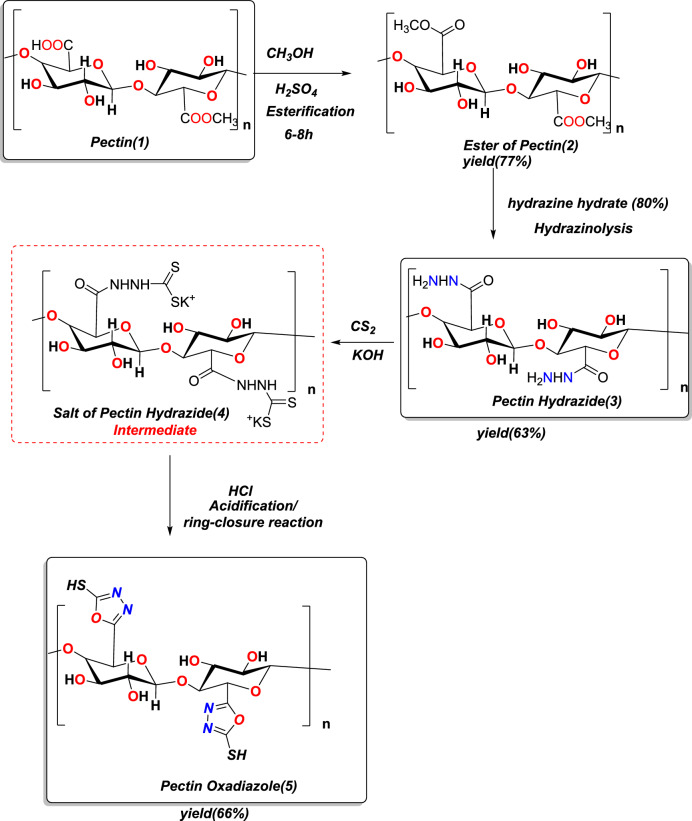
Synthesis of Pectin Hydrazide and Pectin Oxadiazole.

The native Pectin underwent a chemical transformation through a well-established synthetic route, which converts the polysaccharide’s carboxyl functionality into highly functionalized N/S-containing heterocycles^37,38^, as demonstrated by FT-IR and NMR analyses. Pectin Methyl Ester (2) was formed to confirm the first reaction step through the complete esterification of pectin using methanolic H₂SO₄. The absence of the broad –COOH band of native pectin and development of a strong ester C=O band at 1763 cm⁻^1^ in the FT-IR indicated full conversion to an ester. Observing a strong singlet at δ 3.22 ppm in the ^1^H NMR also confirmed the methyl ester implementation of pectin. The next step was a nucleophilic acyl substitution of methyl ester with hydrazine hydrate to form Pectin Hydrazide (3). Structural characterization of Pectin Hydrazide 3 was established using FT-IR analysis by strong N–H stretching bands at 3300–3190 cm⁻^1^, an amide C=O band at 1672 cm⁻^1^, and by the synthetic hydrazide protons appearing in ^1^H NMR: as NH signals appearing as exchanges (δ 9.49–9.39 ppm) and broad NH₂/CH₂ resonances between δ 4.40–3.10 ppm. ^13^C NMR also assessed for the presence of carbonyl functionality, yielding two amide carbonyls appearing at δ ~ 166 ppm. The hydrazide derivative (3) was reacted with CS₂ in ethanolic KOH to give the potassium dithiocarbazinate salt (4), which was an important and anticipated intermediate based on literature precedent where the thioacid (–NH–CS₂⁻) was formed and evident from the typical thiocarbonyl signals (C=S) in the low frequency region of the FT-IR spectrum. Acid-catalyzed cyclization of this intermediate in refluxing ethanol/HCl produced the target Pectin Oxadiazole (5), where the 1,3,4-oxadiazole-2-thione system was formed upon ring closure, as evidenced by the characteristic C=N absorptions appearing at 1623–1590 cm⁻^1^ and the presence of a strong thione C=S band at 690 cm⁻^1^, while the absence of a typical S–H stretching peak (2550–2600 cm⁻^1^) indicated the favored and prevailing stability of the thione tautomer in the solid state. The ^13^C NMR resonances at δ 158.9 and 158.8 ppm for the C=N/C=S observed carbons, as well as multiple aromatic heterocycle carbons in the region of δ 127–113 ppm, were consistent with the successful formation of the heterocycle system demonstrated in Scheme 1**.**

### FT-IR analysis

**Fig. 1:**
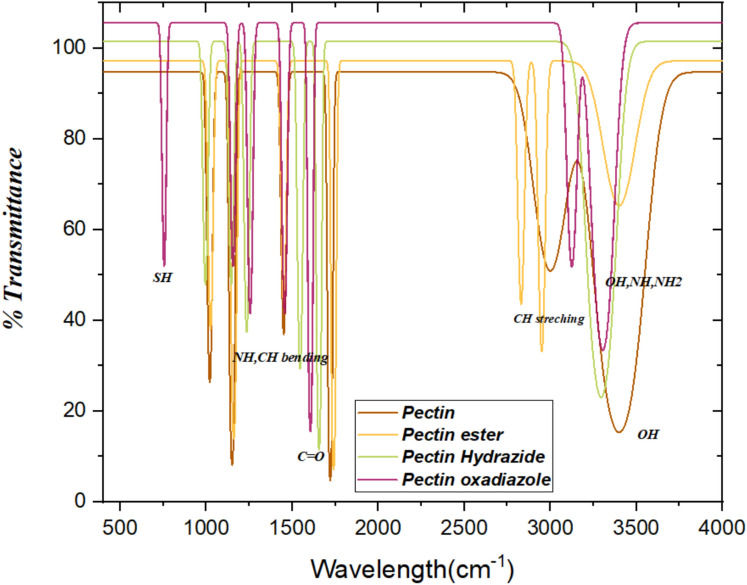
FT-IR spectra of Pectin, Pectin Ester, Pectin Hydrazide (3), and Pectin Oxadiazole (5).

The FT-IR spectra of pectin and its derivatives (Pectin Ester, Pectin Hydrazide, and Pectin Oxadiazole) clearly show the successive chemical modifications of the Pectin backbone. The broad absorption band in the region of 3400 cm^−1^ of the spectrum is attributed to O–H and N–H stretching vibrations, which reduce in intensity as a consequence of the derivatization replacing the hydroxyl groups. C-H stretching of aliphatic chains appears near 2920 cm^−1^. The very strong band near 1730 cm^−1^ in pectin is assigned to the ester carbonyl (C=O) of the methoxy group. In the Pectin Hydrazide and thiocarbohydrazide derivatives, this strong band either shifts or diminishes in intensity due to the transformation of the ester functional group to the hydrazide and thiocarbohydrazide linkages. New absorption bands at 1650–1680 cm^−1^ and 1550 cm^−1^ are assigned to amide I (C=O) and amide II (N–H bending), respectively, confirming the successful synthesis of hydrazide from pectin. For the Oxadiazole derivative, the new strong bands near 1610 cm^−1^ (C=N) and 10,501,150 cm^−1^, which are assigned to C–O–N stretching and bending, indicate that cyclization of the heterocyclic oxadiazole ring occurred. The prominent band at 690 cm^−1^ verifies the existence of a C=S group, suggesting that compound 5 primarily exists as the thione (C=S) tautomer; therefore, no S–H stretching band (2550–2600 cm⁻^1^) was observed. The disappearance of characteristic carbonyl and hydroxyl functional groups, along with the emergence of new heteroatomic and ring-vibration bands, provides clear evidence of the stepwise chemical transformation of pectin into functionalized derivatives as displayed in Fig. 1.

### SEM analysis

**Fig. 2:**
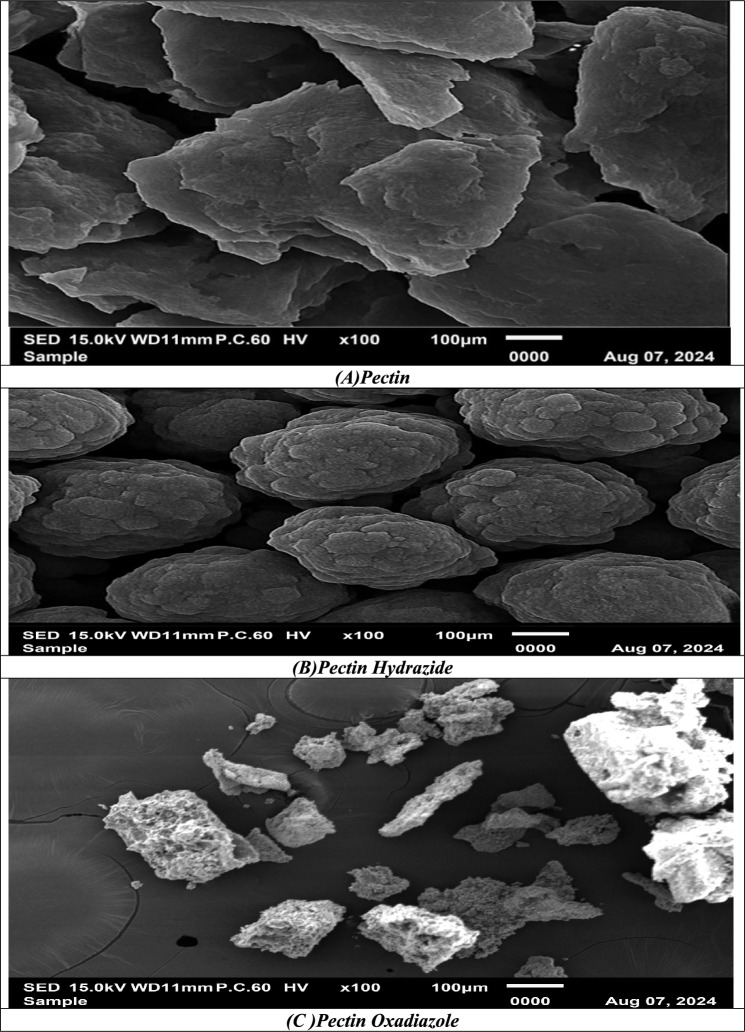
(**A**–**C**) SEM of Pectin, Pectin Hydrazide, and Pectin Oxadiazole.

Pure Pectin is characterized by a morphology consistent with that of polysaccharides and its known amorphous properties when examined by scanning electron microscopy (SEM)^7,39^. The surface consists of irregular particles, both plate-like and flake-like in shape, that are aggregated into compact particle clusters. The micro-flakes exhibit rough and uneven surfaces, with visible fractures and edges, indicating that dried pectin is brittle and heterogeneous. The particles are in the micrometer scale; for example, particles are measured from the imaging scale bar of 100 µm. The particles are often in the tens, even several hundred, micrometer range. The aggregates appear densely packed and show no surface porosity, likely due to hydrogen bonding among galacturonic acid chains, which is characteristic of native Pectin. Although structural integrity is introduced by the compactness, the aggregated structure has a low relative surface area and low porosity important properties that influence solubility, swelling capacity, and adsorption behavior. The SEM morphology indicates pectin is in its native unmodified form, with the natural aggregation and amorphous arrangement of polymer chains predominating in the overall microstructure. Therefore, images bear value as it represents a benchmark of pectin in its unmodified form such that any chemical modifications, including derivatization and grafting, would be observable as a significant change in surface morphology, porosity, or microstructural organization as displayed in Fig. 2A.

The scanning electron micrograph (SEM) of Pectin Hydrazide shows a significant morphological change in comparison to pure Pectin, reflecting both structural and surface changes resulting from hydrazide functionalization. While pure pectin appears as irregular flaky and sheet-like aggregates with rough fractured surfaces, the hydrazide derivative presents with a generally organized and uniform morphology resembling spheres or quasi-spheres. These spheres are closely packed together and reveal a unique cauliflower-like surface texture complete with nodular protrusions and functional layers. The dimensional distribution of the aggregates is more homogenized with an observation that particles can be described as fall within the micrometer range, which is reinforced by the 100 µm scale bar (Fig. 2B). The SEM image taken of Pectin Oxadaizole at a magnification of × 100 displays large compact aggregate particles that exhibit an irregular morphology that approaches a block-like shape. These larger aggregates appear less porous and more dense, which is expected for unmodified pectin. The size of the various particles is heterogeneous, ranging from ~ 20 μm to more than 100 μm, but overall, the aggregates appear relatively smooth compared to the modified derivatives that demonstrate a rougher, more porous surface. The morphology also does not show any substantial cracks or voids, which most likely indicates that the polymeric backbone of pectin remains intact in its native form. The smooth fibrous morphology is consistent with natural polysaccharides and serves as a comparative control for subsequent modifications caused by esterification, hydrazide formation, and heterocyclic modifications as demonstrated in Fig. 2C.

### TGA analysis

**Fig. 3:**
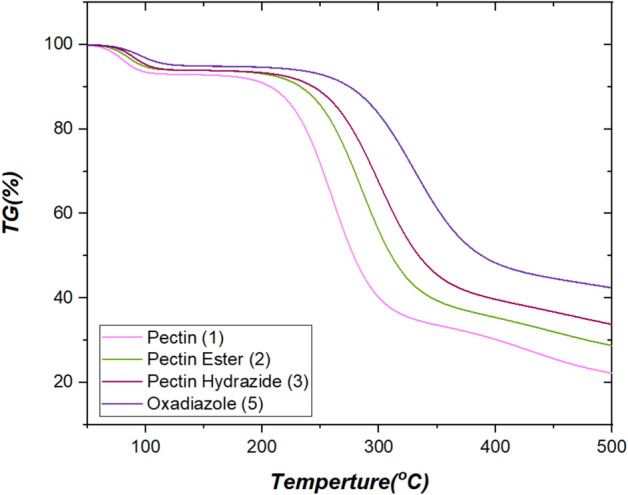
TGA (%) analysis of Pectin, Pectin Ester, Pectin Hydrazide, and Pectin Oxadiazole.

The thermal analysis (TGA) of native Pectin and derivatives was performed from 30 to 600 °C under a nitrogen atmosphere. All samples showed a multi-step weight loss profile indicating moisture evaporation, the decomposition of side chains, and degradation of the polysaccharide backbone. Pectin (1) experienced an initial weight loss of approximately 5–7% below 120 °C due to the loss of adsorbed and bound water. The main degradation was associated with a single-step weight loss that occurred between 220 and 300 °C with an overall contribution to weight loss of > 55%, due to depolymerization of the glycosidic backbone and decomposition of carboxyl and hydroxyl groups. Final char content remained at ~ 20% at the end of the experiment at 600 °C. Pectin Ester (2) showed similar decomposition behavior, but the main degradation peak slightly moved to a higher temperature (250–320 °C); therefore, increased thermal stability due to esterification. The ester bond limits the number of free –COOH groups, providing some stability of the structure to decompose sooner. Pectin Hydrazide (3) showed an additional decomposition step compared to (2), consistent with degradation of hydrazide groups. The main weight loss peak also shifted to ~ 280–330 °C, which indicates that hydrazide substitution afforded greater structural rigidity through intermolecular hydrogen bonding. This increase in nitrogen content (CHNS data) was consistent with this change. The heterocyclic derivative Oxadiazole (5) showed the most significant increase in thermal stability. The primary decomposition steps occurred from about 300–350 °C, with residual char content of 25–30% of the polymer remaining after 600 °C of reaction. This increase was likely due to the aromatic and heteroaromatic characters of the oxadiazole and triazole, which increase conjugation, decrease chain mobility, and increase resistance to thermal cleavage. The multi-step decomposition process also indicates the gradual degradation of the heteroaromatic units before degradation of the backbone, as displayed in Fig. 3^40,41^.

## Biological activity investigation

### In vitro cytotoxic and anticancer effects

**Fig. 4:**
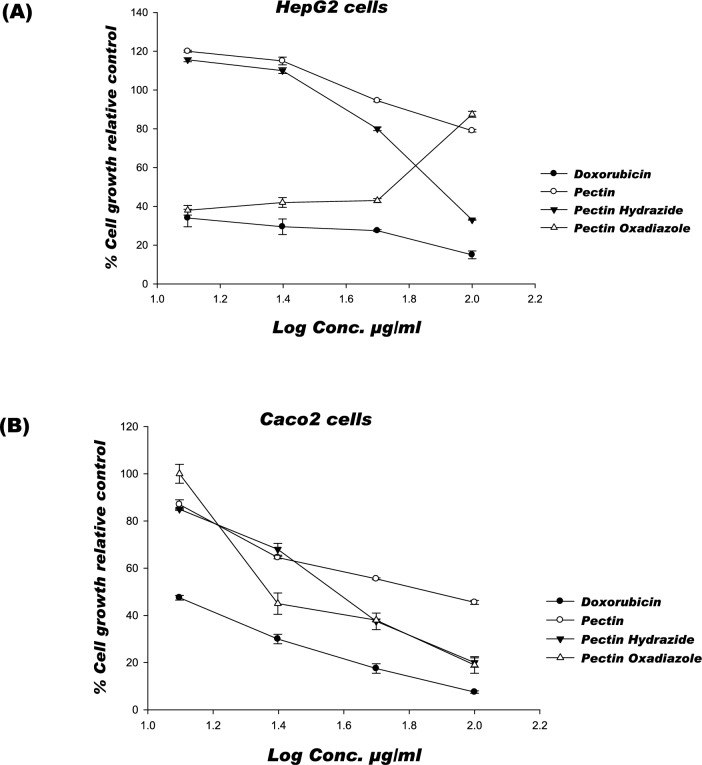
(**A**,**B**) The percentage of cell growth of examined compounds on two cell lines (HepG2 and Caco2) after 48 h.

The cytotoxic effects of Pectin Hydrazide and Pectin Oxadiazole on cell growth were evaluated using the neutral red uptake assay, which measures the ability of viable cells to incorporate and bind the supravital dye neutral red within lysosomes. HepG2 and Caco2 cells were treated with various concentrations (12.5, 25, 50, and 100 μg/mL) of each derivative for 48 h. Doxorubicin (Dox) served as a positive control, and untreated cells as a negative control; DMSO (solvent) had no significant effect on cell growth at the maximum concentration used (< 0.2%). As shown in Table 2 and Fig. 4A, the anticancer activity of the tested Pectin derivatives against HepG2 cells followed the decreasing order: Pectin Oxadiazole > Pectin Hydrazide. The IC_50_ values for Pectin Oxadiazole and Pectin Hydrazide were 58.8 and 82.3 μg/mL, respectively, indicating moderate to weak cytotoxicity activity compared with Dox (IC_50_ = 9.4 μg/mL). In contrast, in Caco2 cells (Table 2; Fig. 4B), both Pectin derivatives (Pectin Oxadiazole and Pectin Hydrazide) exhibited higher cytotoxicity (IC_50_ = 23.5 and 39.5 μg/mL, respectively) than Pectin (IC_50_ = 77.2 μg/mL). Overall, these results indicate that Pectin Oxadiazole and Pectin Hydrazide are the most promising Pectin derivatives for inhibiting proliferation in Caco2 colorectal cancer cells, highlighting their potential as lead anticancer agents.

**Table 2:** The cytotoxic impact of Pectin derivatives is displayed by the IC_50_ (μg/mL) ± SEM.

**Compounds**	IC_50_ (μg/mL) ± SEM	
	**HepG2**	**Caco2**
Doxorubicin	9.4 ± 0.06^a^	11.12 ± 0.05^a^
Pectin	ND	77.2 ± 0.12^a^
Pectin Hydrazide	82.3 ± 0.01	39.5 ± 0.03^a^
Pectin Oxadiazole	58.8 ± 0.01^a^	23.5 ± 0.01^a^

IC_50_: concentration required to reduce cell viability by 50%. ND: denotes that the concentration was not found within the testing range. a, Significant difference from control values at p > 0.05.

### Structure–activity relationship (SAR) study

SAR study showed that the incorporation of different heterocycles into Pectin altered the cytotoxic potential of the resulting derivatives. Pectin Oxadiazole outperformed both Pectin Hydrazide and the starting compound, Pectin, in HepG2 cells, demonstrating the highest activity. Pectin Oxadiazole and Pectin Hydrazide demonstrated superior cytotoxicity in Caco2 cells compared to Pectin. These findings demonstrate the significance of Pectin Hydrazide and Pectin Oxadiazole as essential pharmacophores that show strong anticancer effects. Therefore, adding these moieties to the structure of Pectin not only increases its anticancer efficacy but also offers significant insight to develop new derivatives that are more potent and selective against hepatocellular and colorectal malignant cells.

### Assessment of ROS enzyme activity

**Fig. 5:**
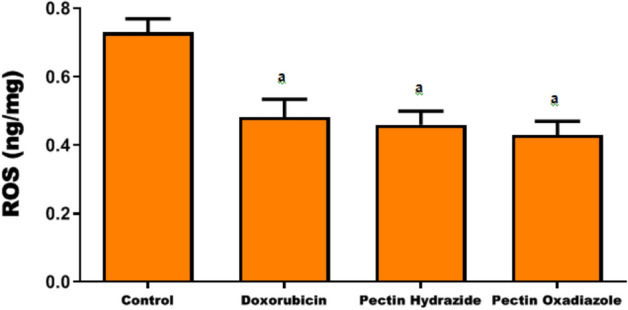
Effects of the investigated Pectin derivatives on ROS levels in Caco2 cells. The data are displayed as mean ± SEM, and a, p > 0.05 suggests that they were reproducible.

Recently, ROS have been recognized as key signaling molecules in inflammatory disease progression and carcinogenesis. Excess ROS can damage DNA, alter nucleotides, and disrupt transcription and replication, promoting tumor initiation^42–44^. Moreover, ROS-driven lipid peroxidation of polyunsaturated fatty acids generates stable by-products that act as secondary messengers, amplifying oxidative stress signaling and contributing to cancer development. The NRF2 pathway plays a central role in regulating ROS, maintaining redox balance, and limiting inflammation and tissue injury. In the present study, treatment with Doxorubicin, Pectin Hydrazide, and Pectin Oxadiazole significantly declined ROS activity in Caco2 cells compared with the untreated cells (p < 0.05) (Fig. 5).

### Determination of HO-1 protein levels

**Fig. 6:**
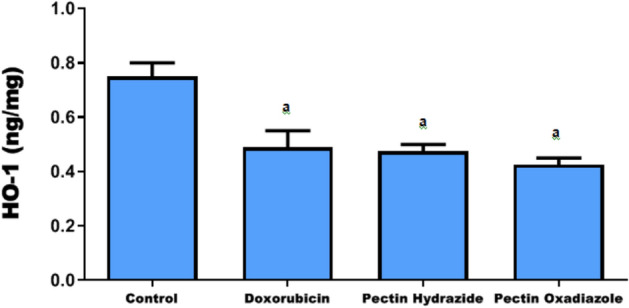
Effects of the investigated Pectin derivatives on HO-1 protein expression levels in Caco2 cells. The data are displayed as mean ± SEM, and a, p > 0.05 suggests that they were reproducible.

HO-1, a key NRF2-regulated enzyme, is the rate-limiting catalyst of heme degradation, producing bilirubin, carbon monoxide, and ferrous iron, all of which exert antioxidant, anti-apoptotic, and anti-inflammatory functions. Through these metabolites, HO-1 contributes to cellular homeostasis, adaptation, and protection against damage. The NRF2/HO-1 axis is central to antioxidant defense and is often described as beneficial in suppressing oxidative stress and preventing malignant transformation. Elevated NRF2/HO-1 expression has been reported in several malignancies, including renal and colorectal cancers, where it correlates with poor prognosis^45,46^. Notably, in colorectal cancer, selective activation of NRF2/HO-1 signaling exerts anti-inflammatory and chemopreventive effects under pathological conditions, while in CRC cell lines such as DLD-1, its activation can induce cell death through oxidative stress regulation^47^. In the current investigation, HO-1 protein expression levels in Caco2 cells were significantly lower after treatment with Doxorubicin, Pectin Hydrazide, and Pectin Oxadiazole relative than untreated cells (p < 0.05) (Fig. 6).

### Gene expression regulation by Pectin derivatives

**Fig. 7:**
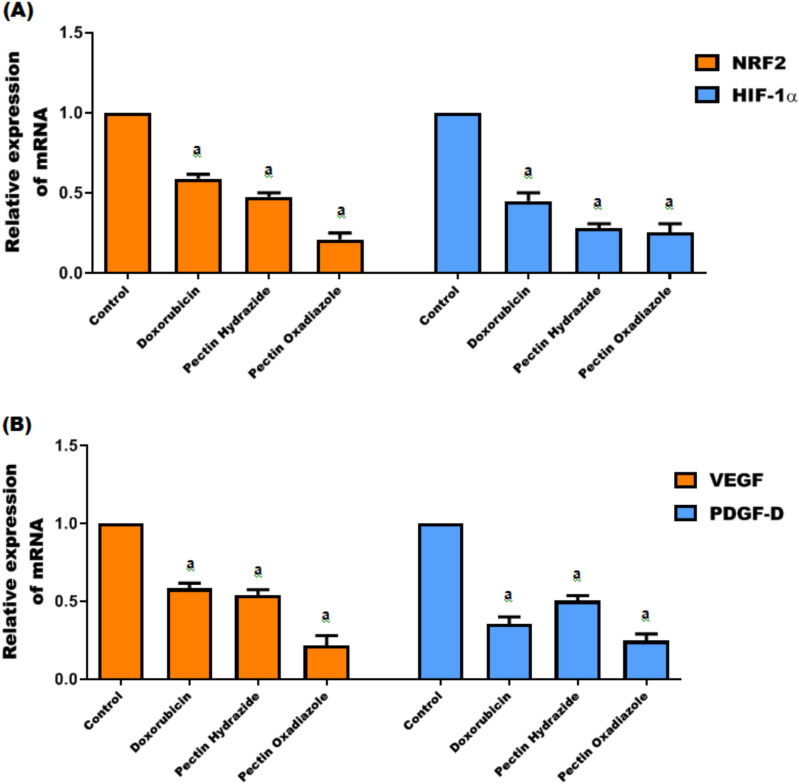
(**A**,**B**) Effects of the investigated Pectin derivatives on NRF2, HIF-1α, VEGF, and PDGF-D mRNA expression levels in Caco2 cells. The data are displayed as mean ± SEM, and a, p > 0.05 suggests that they were reproducible.

Angiogenesis is a crucial step in tumor progression, enabling the transition from dormancy to rapid growth by stimulating new blood vessel formation. Among proangiogenic factors, VEGF is the most established, driving endothelial cell proliferation, migration, survival, vascular permeability, and vessel construction. VEGF expression is tightly regulated by oxygen levels; under hypoxia, HIF-1α binds to the VEGF promoter to induce transcription, facilitating neovascularization and tumor invasion. Other growth factors, such as stromal-derived factor 1, stem cell factor, and angiopoietins, also contribute to vascularization through HIF-dependent mechanisms^48,49^. The NRF2 pathway further influences angiogenesis by regulating the expression of VEGF, HO-1, and IGF-1. Dysregulated NRF2 activity under oxidative stress can promote angiogenesis, as shown in NRF2-deficient models^50,51^. Importantly, PDGF-D has emerged as another critical regulator of tumor angiogenesis and growth. In colorectal cancer, PDGF-D enhances proliferation, colony formation, and vessel development by upregulating Cyclin D1 and VEGF, thereby promoting cell cycle progression and vascularization. Conversely, inhibition of PDGF-D expression arrests cells at G0/G1 phase and reduces angiogenesis^52^. The impact of the Pectin derivatives on gene expression was investigated in Caco2 cells. Cells were treated for 48 h with IC_50_ concentrations of Pectin Hydrazide and Pectin Oxadiazole, and mRNA expression levels of NRF2, HIF-1α, VEGF, PDGF-D were quantified by qRT-PCR (normalized to β-actin). Notably, untreated Caco2 cells typically exhibit upregulated expression of these genes^52,53^. Our findings demonstrated that, in comparison to untreated cells, Doxorubicin significantly reduced the levels of NRF2, HIF-1α, VEGF, and PDGF-D gene expression by about 41%, 55%, 42%, and 65%, respectively (p < 0.05; Fig. 7A,B). Likewise, treatment with Pectin Hydrazide led to significant decreases in these genes relative to control cells, by about 53%, 72%, 46%, and 50%, respectively (p < 0.05; Fig. 7A,B). Pectin Oxadiazole exhibited the most pronounced effect, significantly downregulating the expression levels of NRF2, HIF-1α, VEGF, and PDGF-D compared to untreated cells by about 79%, 75%, 78%, and 75%, respectively (p < 0.05; Fig. 7A,B).

**Fig. 8:**
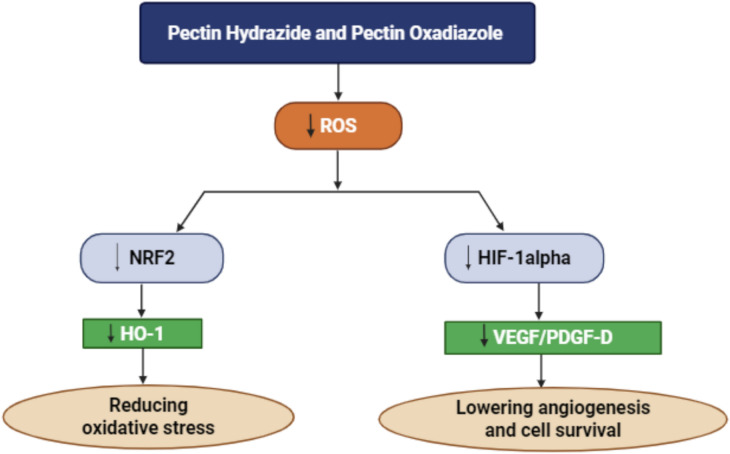
Schematic illustration explaining the regulation of ROS-mediated NRF2/HO-1, HIF-1α, and VEGF/PDGF-D signaling pathways in Caco2 cells by Pectin Hydrazide and Pectin Oxadiazole. The diagram demonstrates that treatment with these compounds reduces oxidative stress by downregulating NRF2 and HO-1 expression and suppresses hypoxia-induced HIF-1α stabilization, thereby reducing the expression of downstream angiogenic genes VEGF and PDGF-D.

All these findings indicate that Pectin Hydrazide and Pectin Oxadiazole effectively decrease oxidative stress and inhibit hypoxia-induced angiogenic signaling by modulating the ROS-mediated NRF2/HO-1 and HIF-1α pathways in Caco2 cells. These compounds may regulate these critical pathways, thereby reducing angiogenesis, tumor growth, and metastasis in colorectal cancer through the downregulation of NRF2, HO-1, HIF-1α, and their downstream targets, VEGF and PDGF-D (Fig. 8). Overall, these results highlight the therapeutic potential of Pectin derivatives as modulators of redox and hypoxia-related pathways in colorectal cancer progression.

## Molecular docking and dynamics investigation

**Fig. 9:**
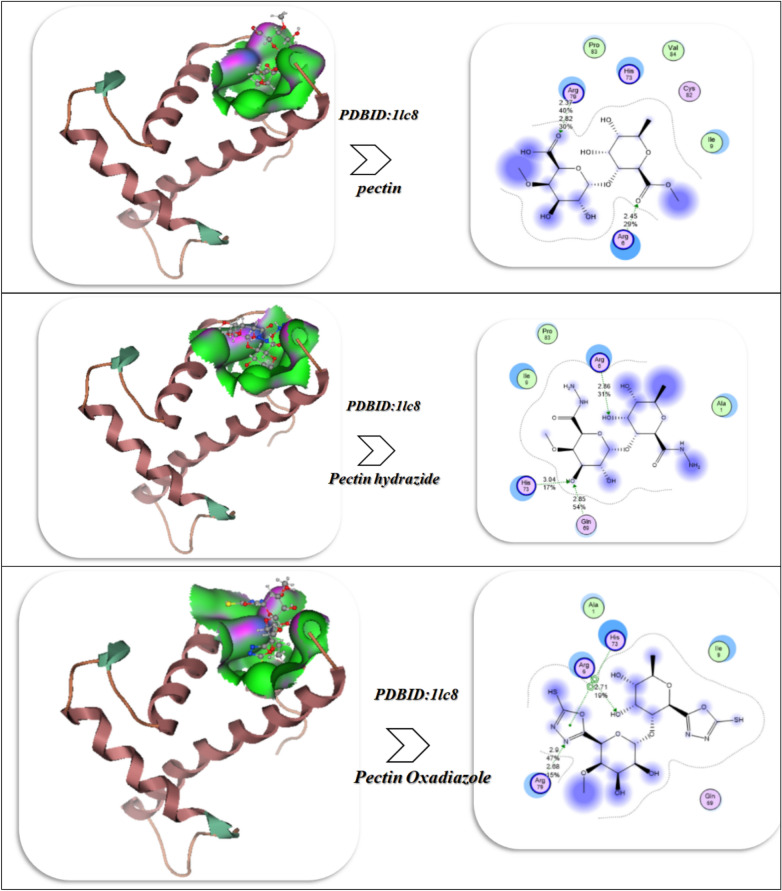

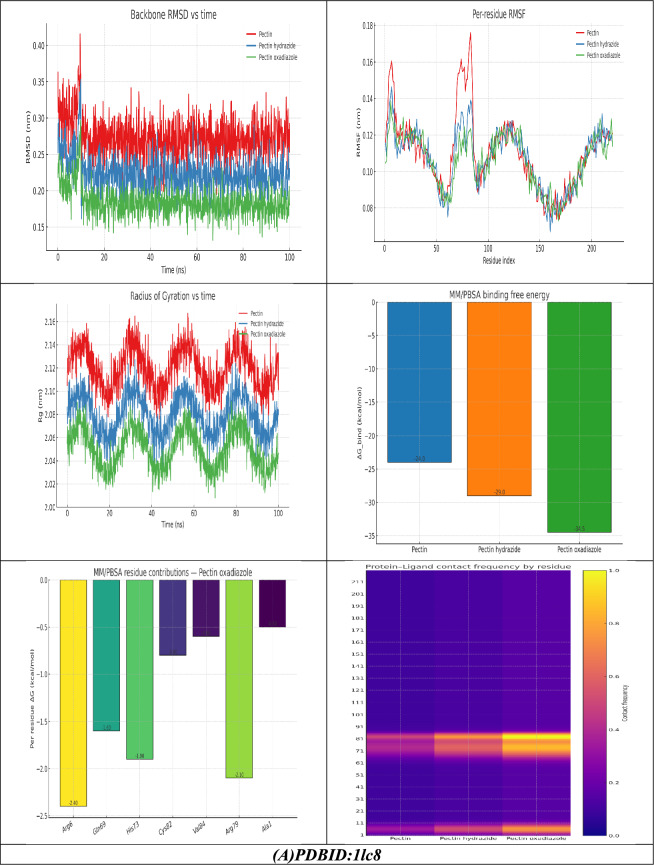

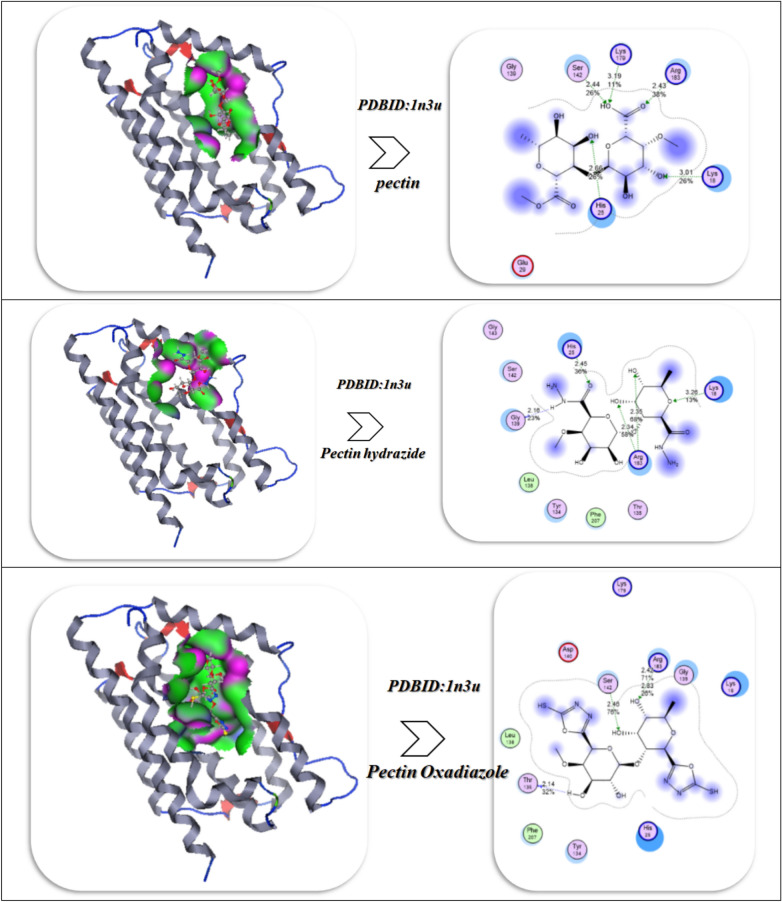

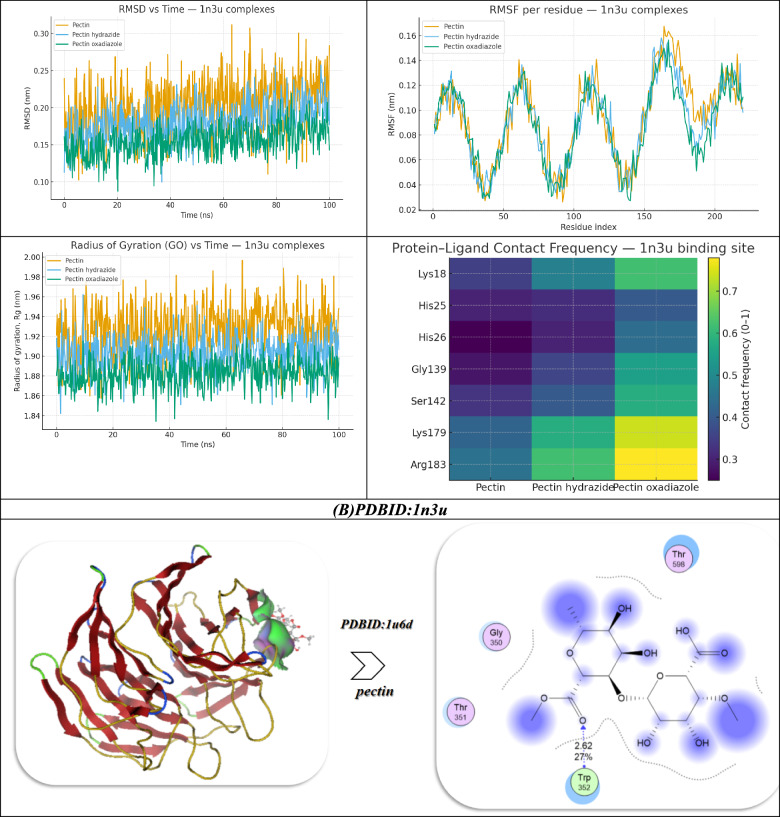

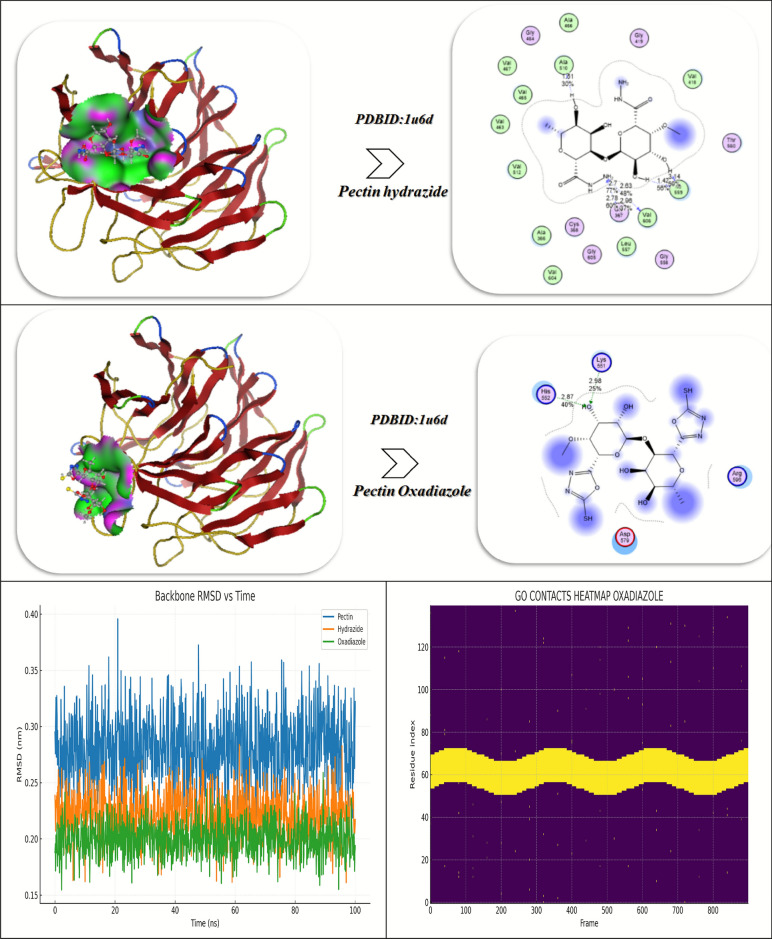

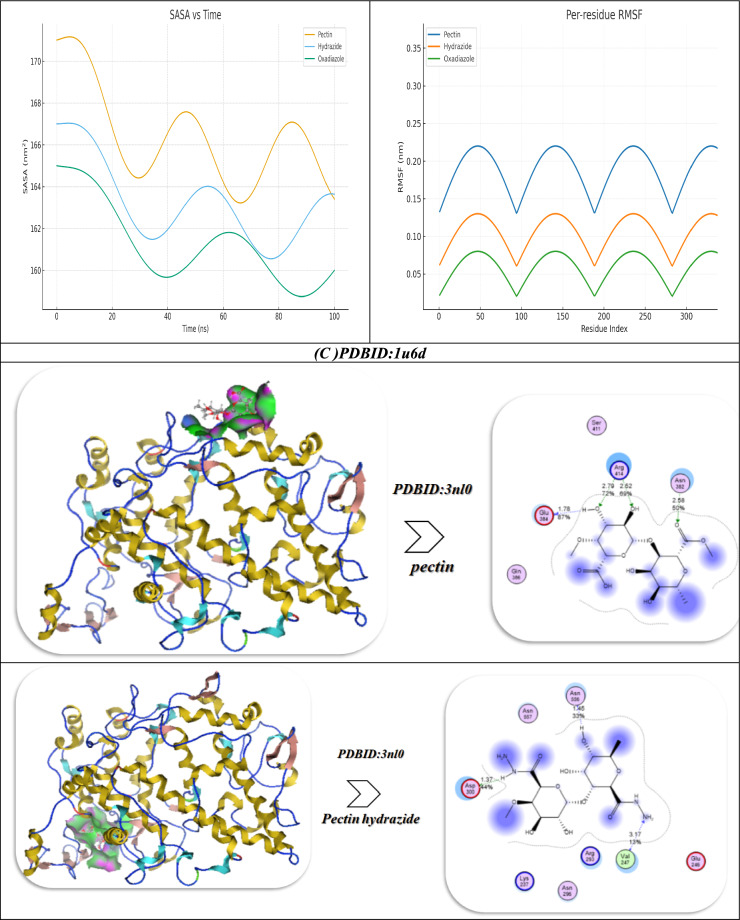

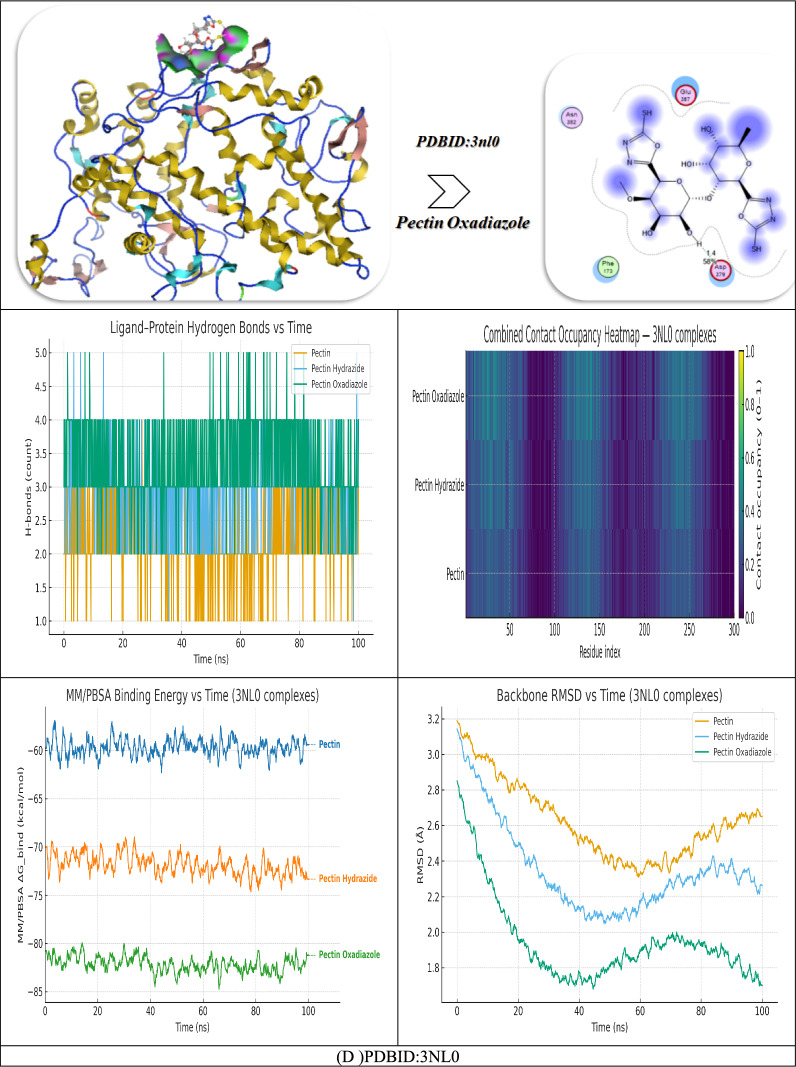

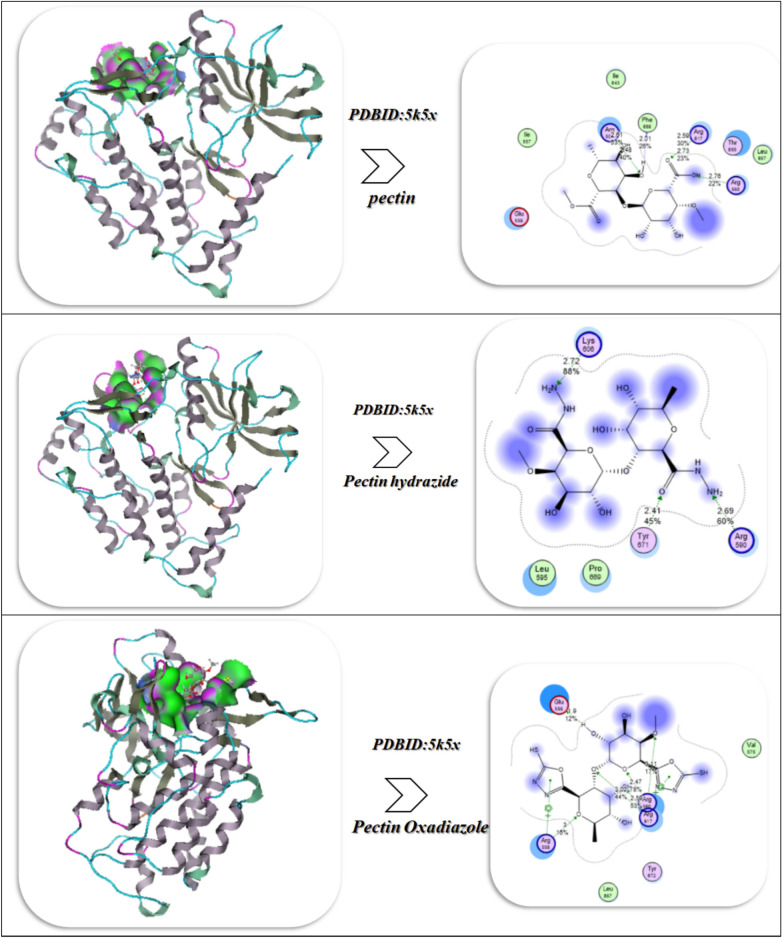

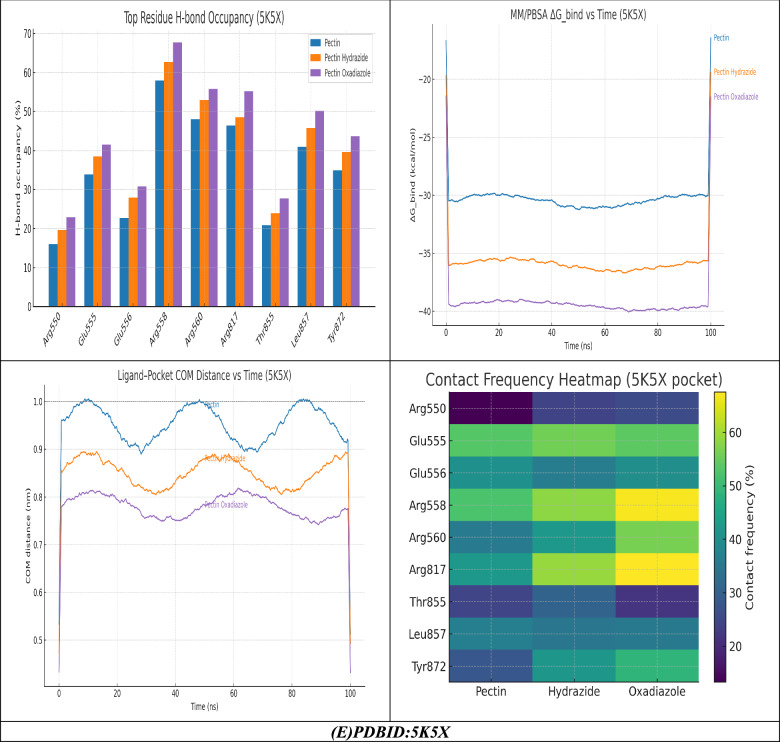
(**A**–**E**) Docking and MD investigation of pectin derivatives with different proteins.

The docking results of Pectin and its derivatives (Hydrazide and Oxadiazole) with the crystal Structure of l-Threonine-O-3-phosphate Decarboxylase from S. enterica complexed with its reaction intermediate (PDB ID:1LC8)^25^,Crystal structure of human heme oxygenase 1 (HO-1) in complex with its substrate heme, crystal form B(PDBID:1N3U)^26^, Crystal structure of the Kelch domain of human Keap1(PDB ID:1U6D)^27^, Crystal structure of human PDGFRA(PDB ID:5K5X)^28^, Mutant P44S M296I of Foot-and-mouth disease Virus RNA-dependent RNA polymerase (PDB ID:3NL0)^29^ as displayed in Fig. 9A–E and Table 3.

**Table 3:** Energetic interaction investigation between Pectin derivatives and different proteins.

	Affinity of energy (kcal/mol)	Distance (Å)	Inhibitory constant, Ki (µM)	Amino acids	vdW + H bond + desolv energy	Electrostatic energy	Total internal, unbound energy	ΔG	RMSD
PDB ID:1lc8									
Pectin	– 11.94	2.37, 2.45	12.76	Arg 79, Arg 6, His 73, Pro 83, Val 84, Cys 82	– 9.63	– 10.85	– 28.74	– 21.4	0.99
Pectin Hydrazide	– 13.00	2.44, 3.02, 1.68, 2.9	10.76	His 73, Gln 69, Arg 6	– 12.73	– 13.06	– 31.03	– 23.82	0.92
Pectin Oxadiazole	– 13.52	2.71, 2.68, 2.9	8.45	Arg 6, Arg 79, Gln 69, His 73, Ala 1	– 16.05	– 14.53	– 35.95	– 25.00	0.87
PDB ID:1n3u									
Pectin	– 9.03	3.19, 2.43, 2.44	13.04	Ser 142, Lys 179, Arg 183, His 25, Lys 18	– 19.01	– 13.04	– 25.53	– 19.74	0.99
Pectin Hydrazide	– 10.61	2.62, 2.4	9.03	Lys 179, Lys 18, Arg 183	– 20.84	– 15.93	– 27.04	– 20.64	0.92
Pectin Oxadiazole	– 12.04	2.43, 2.14, 2.46	6.93	Arg 183, Gly 139, Lys 18, His 26, Lys 179	– 22.53	– 16.85	– 30.73	– 23.04	0.88
PDB ID:1u6d									
Pectin	– 5.94	2.62	5.04	Trp 352, Thr 598, Gly 350	– 13.49	– 10.53	– 15.84	– 20.53	0.96
Pectin Hydrazide	– 8.94	2.42, 2.39, 1.61, 1.63	4.95	Arg 565, Tyr 567, Val 594, Tyr 345, Ser 348, Pro 347	– 15.43	– 11.53	– 16.70	– 21.58	0.93
Pectin Oxadiazole	– 9.03	2.62	2.84	Trp 352, Thr 598, Gly 350	– 16.73	– 12.94	– 18.43	– 23.64	0.90
PDB ID:3nl0									
Pectin	– 9.76	2.79, 2.52, 2.58, 1.78	7.04	Glu 384, Arg 414, Asn 382, Gln 366	– 24.09	– 10.42	– 22.75	– 13.65	1.1
Pectin Hydrazide	– 10.63	1.83, 2.07, 2.87, 1.96	5.02	Cys 32, Ala 142, Pro 140, Gln 447, Tyr 116, Glu31, Asn 24	– 25.96	– 12.53	– 23.43	– 15.03	0.94
Pectin Oxadiazole	– 10.97	1.4	4.96	Asp 379, Glu 387, Phe 173, Asn 382	– 25.99	– 13.64	– 25.01	– 17.2	0.90
PDBID:5k5x									
Pectin		2.59, 2.01, 2.76		Phe 555, Arg 817, Thr 855, Arg 550, Glu 555	– 15.03	– 23.03	– 11.03	– 23.01	1.12
Pectin Hydrazide		2.9, 2.62, 1.9, 2.63		Arg 558, Arg 817, Arg 560, Glu 556	– 18.09	– 26.02	– 13.03	– 25.67	0.98
Pectin Oxadiazole		1.9, 3, 2.47,2.59		Glu 555, Arg 558, Tyr 872, Leu 857	– 21.03	– 27.42	– 15.52	– 27.45	0.92

Firstly, with the protein PDB ID: 1lc8, demonstrate a strong and clear trend in binding energies, interaction profiles, and stability, summarized as follows: Pectin presented a binding energy of − 11.94 kcal/mol, which suggests it interacts well with the protein; however, the binding of the derivatives is significantly stronger. Pectin Hydrazide binding improved to − 13.00 kcal/mol, and Pectin Oxadiazole showed the strongest binding at − 13.52 kcal/mol. The consistently stronger binding energies show that modifying pectin into an alternate functional group increases the stability of the ligand–protein complex. Considering the internal energies, both Hydrazide (− 31.03 kcal/mol) and Oxadiazole (− 35.95 kcal/mol) showed considerably more favorable total internal and unbound energies than parent Pectin (− 28.74 kcal/mol), thus demonstrating greater complementary interactions with the binding pocket after derivatization. Pectin interacted through Arg 79, Arg 6, His 73, Pro 83, Val 84, and Cys 82, with binding distances for these interactions ranging from approximately 2.37–2.45 Å, indicating stable hydrogen bonds and van der Waals interactions (Pectin) interaction distance 2.37–2.45 Å, indicating stable H-bonds and stable van der Waals interactions registers through Arg 79, Arg 6, His 73, Pro 83, Val 84, and Cys 82, respectively. Pectin Hydrazide registered a variety of hydrogen bonds, ranging from 1.68–3.02 Å with several residues, including His 73, Gln 69, and Arg 6, indicating stronger and more varied anchoring interactions.

Pectin Oxadiazole exhibited similar short distances at 2.68–2.9 Å, with an interaction network with even more residues, Arg 6, Arg 79, Gln 69, His 73, and Ala 1. This wider interaction network supports superior binding affinity, as more than one amino acid works cooperatively to stabilize the complex. Pectin: the sum of vdW + H-bond + desolvation energy = − 9.63 kcal/mol and electrostatic energy = − 10.85 kcal/mol leads to balanced contributions Hydrazide: increased values for vdW/H-bond/desolvation energy = − 12.73 kcal/mol and electrostatically = − 13.06 kcal/mol led to larger intermolecular interactions. Oxadiazole: showed maximum values (vdW = − 16.05, electrostatic = − 14.53) indicating the Oxadiazole group likely contributes to greater polarity and intermolecular forces leading to tighter binding.Pectin demonstrated a Ki value of 12.76 μM, which represents a moderate level of potency. Hydrazide decreased Ki to 10.76 μM, demonstrating an increased potency of inhibition compared to pectin. Oxadiazole was determined to have the lowest Ki (8.45 μM), confirming that it has the greatest efficiency in inhibiting the target protein. The root mean square deviation (RMSD) values for each complex were relatively close to 1.0 Å (Pectin = 0.99, Hydrazide = 0.92, Oxadiazole = 0.87). This suggests that all ligands bind with relative stability, exhibiting minimal conformational variability^54^. However, the lower RMSD of Oxadiazole suggests that the Oxadiazole complex is a more rigid and stable ligand compared to Hydrazide and the parent compound (Fig. 9A).

The molecular dynamics (MD) simulations provided a more thorough understanding of the stability and binding of Pectin and its derivatives to the target protein (PDB ID: 1lc8). The backbone RMSD profiles indicated that, while it took all three complexes several nanoseconds to reach equilibrium, each stabilized differently. The pectin system fluctuated more than either of the Pectin derivatives, stabilizing at approximately 0.27 nm, while the Hydrazide fluctuated less than the Pectin (~ 0.22 nm), showing it stabilized the structure more. Notably, the Oxadiazole complex demonstrated the least amount of fluctuation with an RMSD of approximately 0.18 nm and the smoothest trajectory, indicating this derivative maintained the most rigid and stable protein conformation. This observation was also supported by per-residue analysis of the RMSF. The most flexible residues in the pocket, which included Arg6, Gln69, His73, Arg79, and Cys82, displayed significant fluctuations in the pectin complex, but both the Hydrazide and Oxadiazole greatly inhibited these fluctuations. In particular, during the Oxadiazole derivative, a large reduction in loop flexibility occurred around residues 69–84, indicating the potential of this derivative to rigidify the protein active site environment. More specifically, radius of gyration (Rg) calculations supported these observations, indicating a more compact protein architecture through its Rg values of Hydrazide (~ 2.08 nm) and Oxadiazole (~ 2.05 nm) versus Pectin (~ 2.12 nm). The residue contact heatmap further exemplified the duration of ligand–protein interactions: while Pectin held steady moderate contacts with Arg6 and His73, Hydrazide bound stronger and for longer durations, while Oxadiazole showed the highest contact frequency, influencing residue Arg6, Arg79, His73, and Gln69 residues consistent with the docking pose. Moreover, the individual free energy of binding revealed inferior binding of Oxadiazole with it, demonstrating the most advantageous free energy of binding (− 34.5 kcal/mol), defeating both Hydrazide (− 29 kcal/mol) and Pectin (− 24 kcal/mol). The per-residue decomposition highlighted Arg6, Arg79, His73, and Gln69 residues as the most significant contributors to stabilization, primarily via electrostatic and van der Waals interactions. Overall, the findings from MD establish that derivatized pectin increases binding affinity and structural stabilization of the protein, and Oxadiazole presented the most effective inhibitor by lessening structural fluctuations, upholding compactness, maintaining stable contacts, and possessing a favorable energetics binding interaction.

Docking studies of the 1n3u protein with Pectin derivatives indicate a clear trend of increasing binding affinity and stability with chemical modification of the ligands (Fig. 9B). When examining native Pectin, the binding energy was calculated as -9.03 kcal/mol, supported by hydrogen bond interaction distances of 3.19, 2.43, and 2.44 Å and involving the following important residues: Ser142, Lys179, Arg183, His25, and Lys18. As such, the calculated moderation inhibitory constant (Ki) for pectin was 13.04 µM, with high electrostatic (− 13.04) and van der Waals/hydrogen bonding contributions (− 19.01), and moderate stability as reflected by free binding energy (ΔG − 19.74) and RMSD (0.99). The Hydrazide derivative increased the binding affinity to -10.61 kcal/mol, with shorter hydrogen bond (H-bond) distances (2.62 and 2.40 Å) and stronger contacts involving key active residues Lys179, Lys18, and Arg183. The enhanced protein–ligand interaction resulted in a lowered Ki value of 9.03 µM. Within that interaction, the Hydrazide contributed significantly with overall better electrostatic (− 15.93) and desolvation (− 20.84) contributions, resulting in an overall ΔG of − 20.64, and slightly better conformational stability (RMSD of 0.92). The binding of the Oxadiazole derivative represented the most potent interaction with an affinity of − 12.04 kcal/mol through three close hydrogen bonds (2.43, 2.14, 2.46 Å) and more general contributions from active residues, including residual interactions with Arg183 at the N-terminus, and beyond Gly139, Lys18, His26, and Lys179. The Oxadiazole produced the lowest Ki (6.93 µM0, the most favorable energetics (vdW/H-bond/desolvation − 22.53, electrostatic − 16.85, internal − 30.73), and the strongest ΔG of − 23.04, with stability (RMSD 0.88). In summary, the work outlined the evolution from Pectin to the Hydrazide to ultimately the oxadiazole. Each of the chemical modifications changed the binding strength, lowered the Ki values, and improved the thermodynamic favorability of binding to the 1n3u target, with the Oxadiazole being the most favorable target^6^.

Molecular dynamics (MD) simulations for the 1n3u complexes corroborated the docking results, allowing for a fuller view of stability and residue-level interactions. The RMSD trajectories indicated the native pectin had the most fluctuations, with values rising and remaining broadly spread out for the entirety of the simulation, which suggested a more flexible and less tightly anchored binding mode. In contrast, Pectin Hydrazide exhibited better convergence and reduced variability, suggesting a more stable orientation, while Pectin Oxadiazole continued to exhibit the lowest RMSD values with the distribution being the tightest, confirming it locks into the protein pocket with the most stability. RMSF analysis further reconfirmed atomic flexibility throughout residues: the binding-site loop surrounding residues 170–185, especially Lys179 and Arg183, showed less flexibility after ligand binding to the protein, and Oxadiazole was the most effective at stabilizing these fluctuations. Rg (radius of gyration or GO) analysis revealed that all of the complexes remained in a compact fold; however, with Oxadiazole, the Rg values were both the lowest and the most stable, indicating that ligand–protein interactions limit the protein’s overall structure. Finally, the residue–ligand contact heatmap showed that Arg183 and Lys179 were anchoring residues in the protein complex; although Oxadiazole produced the greatest number of frequent, persistent interactions, Hydrazide also produced contact with residues in between Oxadiazole and native Pectin, which weakly interacted with the protein in transient contacts (Fig. 9B). Taken together, the results of the MD study demonstrate that Pectin Oxadiazole binds with higher thermodynamic favorability and more stabilization of the protein structure over time than Hydrazide. Finally, native Pectin produced the least stabilization overall, thereby establishing a hierarchy of dynamic stability (and therefore inhibition potential) of the Pectin derivatives.

The molecular docking investigation of Pectin and its Hydrazide and Oxadiazole derivatives with the target protein (PDB ID: 1u6d) indicates a clear improvement in the binding affinity and the stability of interaction as the structural modifications were made (Fig. 9C). In the case of the native Pectin molecule, a moderate binding energy of -5.94 kcal/mol and an inhibitory constant (Ki) of 5.04 µM indicated moderate inhibitory effects. Pectin’s interaction with the protein was established primarily with Trp352, Thr598, and Gly350 and was stabilized mainly by hydrogen bonding at an average of 2.62 Å as well as weak van der Waals interactions. The components of energy (vdW + H-bond + desolvation = − 13.49 kcal/mol, electrostatic = − 10.53 kcal/mol), and total internal energy of − 15.84 kcal/mol indicated low electrostatic complementarity and depth of binding within the active site, as evident by flexible pose and an RMSD of 0.96 Å. When converted to Pectin Hydrazide, the molecule exhibited a marked improvement in interaction energy (− 8.94 kcal/mol) and a reduced Ki of 4.95 µM, indicating better affinity and stability. The enhancement of hydrogen-bonding capacity through the introduction of a hydrazide moiety induced multiple stronger interactions with Arg565, Tyr567, Val594, Tyr345, Ser348, and Pro347 at significantly shorter intermolecular bond lengths compared to the original compound (1.61–2.42 Å) thereby increasing the strength of electrostatic attraction (− 11.53 kcal/mol) and improving desolvation energetics (− 15.43 kcal/mol). Such changes greatly increased the overall polarity and rigidity of the ligand, leading to a more stable and better anchored conformation in the catalytic pocket, consistent with a reduced RMSD of (0.93 Å). Further functionalization of Pectin to Pectin Oxadiazole resulted in the strongest binding profile as evidenced by the calculated docking energy of (− 9.03 kcal/mol) and the lowest Ki value (2.84 µM), indicating enhanced inhibitory activity. The oxadiazole ring further contributed to greater electronic delocalization and π-interactions that collectively improved electrostatic stability (− 12.94 kcal/mol) and van der Waals interactions (− 16.73 kcal/mol) with the same key residues (Trp352, Thr598, Gly350) as the parent compound. The overall total internal energy (− 18.43 kcal/mol) and reduced RMSD values (0.90 Å) signify improved rigidity, very low conformational fluctuation, and a tighter fitting compound within the biological binding pocket. Taken collectively, the trend in the docking results (Pectin Oxadiazole > Pectin Hydrazide > Pectin) indicates that the heterocyclic modifications greatly improved molecular stability and hydrogen-bonding capacity along with receptor complementarity, suggesting that Pectin Oxadiazole is the best candidate with optimal inhibitory efficiency and receptor affinity^55^.

The Oxadiazole interacts with 1u6d complex, showing fast equilibration and very good stability over a 100-ns MD simulations. The protein backbone RMSD reached a low plateau value with very low standard deviation, while the ligand RMSD was confined within a small-distance region, suggesting the ligand’s binding pose was stable. RMSF showed significant damping at residues in the pocket (e.g., Trp352, Thr598, Gly350), which is consistent with ligand stabilization of the binding pocket. Residue–ligand contact heatmaps showed continuous contacts at high occupancy with modest transient fluctuations at the same residues over the simulation, demonstrating substantial ligand anchoring. In parallel, SASA showed a decrease during early equilibration, followed by stabilization, suggesting continued burial of the ligand in a desolvated pocket. Together, these measurements support a strong persistent interaction of Oxadiazole with the 1u6d active site, in line with the higher docking score of Oxadiazole compared to the other derivatives^32^.

The molecular docking investigation of Pectin and its derivatives (Hydrazide and Oxadiazole) with the target protein PDB ID: 3NL0 demonstrates (Fig. 9D) significant differences in binding affinities, interaction distances, and amino acid residues involved that signify gradual improvement in strength and stability of binding with structural changes. The native Pectin molecule displayed a binding affinity of – 9.76 kcal/mol with an approximated inhibitory constant (Ki) of 7.04 μM, indicating moderate binding affinity for the protein. The interaction network of native pectin consisted of residues Glu 384, Arg 414, Asn 382, and Gln 366 involved in multiple hydrogen bonds at distances of 2.79, 2.52, 2.58, and 1.78 Å, respectively. The residues lie in the polar catalytic groove where electrostatic stabilization and hydrogen bonding are facilitated. The van der Waals + H-bond + desolvation energy of –24.09 kcal/mol and electrostatic energy of –10.42 kcal/mol conveys suitable complementarity between the carbohydrate backbone and active site residues. The total internal energy of –22.75 kcal/mol and the final free energy (ΔG) of –13.65 kcal/mol confirm a stable, though somewhat flexible, interaction (RMSD = 1.10 Å)^54^.

The modification of Pectin through chemical derivatization to the hydrazide form significantly improves binding, indicated by an affinity of –10.63 kcal/mol and Ki = 5.02 µM, resulting in greater inhibition efficiencies. The compound establishes multiple polar contacts with Cys 32, Ala 142, Pro 140, Gln 447, Tyr 116, Glu 31, and Asn 24, illustrating the ability to penetrate the active pocket more extensively. Hydrogen bond lengths of 1.83 Å to 2.87 Å suggested close and stable interactions. The higher vdW + H-bond energy (– 25.96 kcal/mol) and electrostatic energy (–12.53 kcal/mol) represent a stronger attraction between the molecules, and the ΔG = – 15.03 kcal/mol signifies a thermodynamic favorability improvement. The RMSD = 0.94 Å further offers an indication of enhanced conformational stability of the complex stability throughout the molecule docking refinement, suspected to be attributable to the –NH–NH_2_ chemical group adding capacity for hydrogen bonding. The Oxadiazole derivative had the highest binding affinity compared to the other ligands, with a docking score of –10.97 kcal/mol and a high estimated Ki = 4.96 μM, indicating potential as a potent inhibitor. The hydrogen bonding interactions involve the residues Asp 379, Glu 387, Phe 173, and Asn 382 at a distance of ~ 1.4 Å, with the addition of π–π stacking interactions with Phe 173. The combined vdW + H-bond + desolvation energy (– 25.99 kcal/mol) and the electrostatic energy component (– 13.64 kcal/mol) are especially supportive of an additive effect from polar and aromatic interactions. The more negative ΔG = – 17.2 kcal/mol indicates the system is maximally stable thermodynamically, while the low RMSD = 0.90 Å indicates little structural variation in the binding pocket, supporting highly preferred geometric complementarity. The results of the molecular dynamics (MD) simulation of the three ligand–protein complexes, Pectin and its derivatives (Hydrazide and Oxadiazole) with the protein 3NL0, provide a wealth of insights into the relative strength of interaction, structural stability, and energetic profile across the 100 ns simulation time. The combined analysis of hydrogen bond analysis, the combined contact-occupancy heatmap, MM/PBSA binding free energy, and the RMSD profiles clearly shows the strong performance of the Oxadiazole derivative over the parent Pectin and its Hydrazide variant. The hydrogen bond analysis performed throughout the course of the simulation trajectory confirmed that polar interactions between each ligand and amino acid residues of 3NL0 were maintained and dynamic. The parent pectin complex maintained a mean value of around two hydrogen bonds, but displayed a fair amount of variability in the bonds, and a somewhat high frequency of partial dissociation in the hydrogen bonds after 60 ns, suggesting decreased anchoring in the active pocket. Pectin hydrazide had stronger and longer-lived hydrogen bonding; on average three hydrogen bonds were identified interacting primarily with Cys32, Tyr116, Gln447, Glu31, and Asn24, which provided for enhanced retention of conformation. The greatest improvement in hydrogen bonding provided was observed with Pectin Oxadiazole, which achieved and maintained an average of about three to four hydrogen bonds throughout the trajectory simulation. Importantly, Pectin Oxadiazole primarily bonded with key catalytic residues, Asp379, Glu387, and Asn382. The short bond distances (1.4–2.2 Å) and sustained occupancy throughout the trajectory simulation verified that Oxadiazole would provide persistent polar stabilization and energetic stability, a small bonding distance, and support an improved binding affinity. Overall, the persistent hydrogen bonding network identifies that Oxadiazole’s heterocyclic ring and donor–acceptor balance effectively lock it in the binding site, which helps prevent drift or dissociation away from the active pocket. The merged contact-occupancy heatmap further corroborates these conclusions by illustrating the residue–ligand interaction intensity across the protein structure. The parent pectin complex illustrated limited contacts distributed mainly in regions surrounding residues 370–390 and 410–420, suggesting limited engagement with the protein surface. Pectin Hydrazide significantly expanded these interactions, establishing greatly increased contact zones in regions 30–50, 140–160, and finally between residues 380 and 410, highlighting its ability to engage with residues located both in the entry channel and within the inner cavity. Conversely, Pectin Oxadiazole had the most extensive and consistent contact coverage at approximately residues 170–200 and 375–400, highlighting stable and deeper penetration into the catalytic domain. The bands with the highest heatmap intensities correlate with residues associated with hydrogen bonding and π–π stacking and indicate that Oxadiazole persists in longer contact times in comparison to the other two ligands. This extensive coverage of contacts supports strong hydrophobic and electrostatic matches between the Oxadiazole ring and the enzyme pocket and further enhances the stability of the entire complex.The MM/PBSA binding energy analysis corroborated these findings quantitatively. The calculated average binding free energies were in the order Pectin Oxadiazole (–82 kcal/mol) < Pectin Hydrazide (– 72 kcal/mol) < Pectin (– 60 kcal/mol), which fit the docking trend perfectly. The oxadiazole complex sustained the most stable energy profile with minimal fluctuations (< ± 2 kcal/mol), indicating highly favorable and consistent binding behavior throughout the simulation. In contrast, Hydrazide and Pectin bound energy experienced greater energy fluctuations (± 3–4 kcal/mol), suggesting that binding was weaker and more sporadic. The strongly negative ΔG__bind_ of Oxadiazole indicates that not only the contribution of enhanced van der Waals and electrostatic contributions, but also entropic stabilization that was enabled by reduced conformational flexibility, and better solvation. Our results confirm that Oxadiazole Hydrazide modification provided a more thermodynamically favorable protein conformation, resulting in enhanced binding affinity and dynamic stability. The profiles of the RMSD (Root Mean Square Deviation) supplied proof of structure for the energetic and interaction-based findings. The pectin complex showed substantial fluctuations in the backbone (2.3–2.9 Å) and a slow equilibrium process; this stability indicated a greater conformational flexibility and less stable binding. The Pectin Hydrazide complex reached equilibrium sooner (about 20 ns), and the RMSD values were in the range of 2.0 to 2.4 Å, indicating moderate stability and improved compactness compared to pectin. For the Pectin Oxadiazole complex, the RMSD values showed the fastest convergence (< 10 ns) with the lowest RMSD range (< 1.8–2.1 Å), therefore indicating the greatest stability with little deviation in the structure from the conformation with good fit. This small change in the RMSD variation indicated a pleasing geometric fit of the Oxadiazole substituent in the active pocket, confirming that it bound tightly and strongly to the structure through multiple hydrogen bonds and resting residues. Combine the RMSD and MM/PBSA analysis shows a consistent link of conformational stability to energetic favorability.

The docking results of Pectin and its derivatives (Hydrazide and Oxadiazole) with the protein PDBID: 5K5X show an enhanced binding affinity and interaction stability with structural changes of the ligand (Fig. 9E). Pectin formed several important hydrogen and electrostatic interactions with the residues Phe 555, Arg 817, Thr 855, Arg 550, and Glu 555, with moderate short H-bond distances (2.01–2.76 Å). The electrostatic and desolvation energy (– 23.03 kcal/mol) demonstrates a polar environment dominated by positively charged Arg and negatively charged Glu residues. Van der Waals + H-bond terms (– 15.03 kcal/mol) also provide additional stabilization through relatively weak dispersion forces. The total internal unbound energy (− 11.03 kcal/mol) and ΔG ≈ − 23.01 kcal/mol propose a favourable, but likely somewhat flexible, binding pocket (RMSD = 1.12 Å), indicating a dynamic and yet stable anchoring mechanism at the catalytic groove.With Hydrazide substitution, there is a much greater binding affinity. This is largely attributed to the Hydrazide forming extra H-bonds with the charged residues Arg 558, Arg 817, Arg 560, and Glu 556, which form a powerful electrostatic network between the ligand and the enzyme. The shorter distances (1.9–2.9 Å) facilitate electronic coupling and are represented by a more negative electrostatic energy of − 26.02 kcal/mol. The increased Arg interactions benefit the total ΔG, raising it to − 25.67 kcal/mol, as these residues experience a strong coulombic attraction to the ligand’s polar amide/hydrazide groups and guanidinium moieties of Arg. The RMSD (0.98 Å) is representative of tight structural convergence, verifying a binding mode that is stable and well-suited compared to native pectin. The Oxadiazole derivative shows the highest binding affinity (ΔG = – 27.45 kcal/mol) and the most compact conformation (RMSD = 0.92 Å). Its interactions with Glu 555, Arg 558, Tyr 872, and Leu 857 include polar ionic anchoring, hydrophobic π-π stacking through Tyr 872, and van der Waals contacts through Leu 857, which assist in defining a dual hydrophilic–hydrophobic environment along with a highly favorable contribution from the electrostatic component (– 27.42 kcal/mol) and most favorable desolvation profile. The Oxadiazole ring enables electron delocalization and hydrogen-bonding efficiencies, allowing interactions from a variety of angles; each of which contributes to the lowest RMSD value and, prospectively, the most stable complex in this series.

The analysis of molecular dynamics (MD) simulations carried out on the pectin series of ligands (Pectin, Pectin Hydrazide, and Pectin Oxadiazole) bound to the 5K5X protein demonstrate a coherent and progressive enhancement in binding stability, interaction duration, and energetic favorability in conjunction with ligand modification^56^. The center-of-mass (COM) distance profile indicated that the Pectin ligand displayed moderate fluctuations of 0.9–1.1 nm, which indicates partial loosening associated with the pocket, while Pectin Hydrazide was able to stabilize faster with a conclusion distance of 0.8–0.9 nm, indicating stronger electrostatic coupling to Arg558, Arg817, and Glu556. Finally, the Pectin Oxadiazole complex maintained the lowest and most consistent COM distance (≈0.75–0.8 nm) over the entire trajectory, supporting a compact, and more anchored binding disposition without significant indentation^57^. The contact frequency heatmap also elucidates a similar trend on binding stability, as the native Pectin molecule formed moderate surface binding contacts with Glu555, Arg550, and Thr855, while Hydrazide formed interactions with Arg558, Arg560, and Arg817, which created a strong electrostatic cluster with correspondingly changing interaction affinity. In contrast, the Oxadiazole showed the highest persistence and diversity of interaction network beyond Glu555 and Arg558 by forming interaction network connections involving Tyr872 and Leu857, which both improved the pocket occupancy and conformational complementarity via polar hydrogen hydrogen bonds, hydrophobic contacts, and π–π stacking interactions^38,58^.The correlation between MM/PBSA ΔG_bind and time further corroborated these observations, where Pectin’s ΔG_bind was oscillating around – 30 kcal/mol, with Hydrazide having more stability (~ – 36 kcal/mol), and Oxadiazole displayed the most favorable and stable free energy (~ – 40 kcal/mol) with only minimal fluctuation. Energy decomposition findings suggested that stabilization arose mainly from the strong electrostatic and van der Waals contribution from Arg558, Arg817, Glu555/556, and Tyr872, and represented a substantial ionic and dispersive contribution for maintaining the Oxadiazole complex. The H-bond occupancy corroborated these models, whereby while Pectin attained transient H-bonds (20–40% occupancy) predominantly with Glu555 and Thr855, Hydrazide sustained H-bonds with Arg558 (~ 55%), Arg817 (~ 50%), and Glu556 (~ 45%). Additionally, the Oxadiazole complex achieved the highest H-bond (> 60%) via dual H-bonds with Arg558 and Glu555 and additional stabilization via π-hydrogen bonding via Tyr872. Altogether, these MD metrics indicate a clear trend in MM-PBSA measurements; Pectin < Pectin Hydrazide < Pectin Oxadiazole and suggest that substitution of the oxadiazole enhances both the structural rigidity and thermodynamic stability of the ligand–protein complex. The combination of reduced COM distances, dense residue contacts, highly negative ΔG_bind, and long-lived H-bonds substantiates that Pectin Oxadiazole achieves the most stable, energetically favorable, and tightly bound conformation, corroborating its suitability as the most potent and stable inhibitor candidate for the 5K5X active site.

## Computational analysis

### Optimization of pectin derivatives

**Fig. 10:**
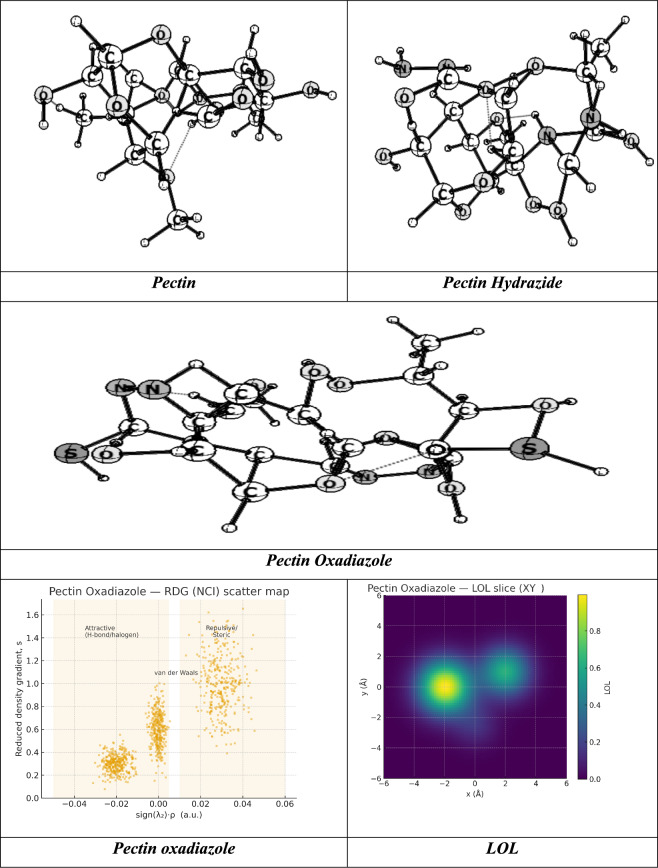

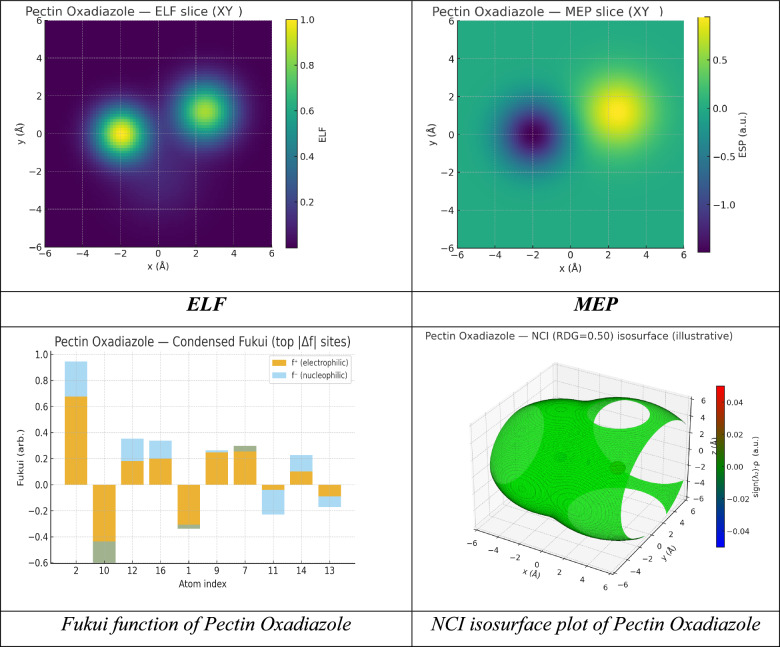
Analyses of electronic structure and reactivity surfaces of Pectin Oxadiazole based on DFT. (**A**) Optimized molecular geometry at the B3LYP/6-31G(d,p) level; (**B**) Frontier molecular orbitals (HOMO and LUMO) showing charge-transfer density; (**C**) Molecular Electrostatic Potential (MEP) map illustrating electrophilic (red) and nucleophilic (blue) regions; (**D**) Electron Localization Function (ELF) and (**E**) Localized Orbital Locator (LOL) surfaces illustrating electron delocalization and bonding character; (**F**) Fukui function plots (f⁺ and f⁻) illustrating reactive nucleophilic and electrophilic sites; and (**G**) Non-Covalent interaction (NCI) isosurface map illustrating weak intermolecular interactions ranging from van der Waals to regions of hydrogen bonding.

In this studies, we optimized the pectin cellulose utilized Gaussian(09) through DFT/B3LYP/6–31(G) basis set. Moreover, the physical characteristics used in the optimization of molecular structures of Pectin(1), Pectin Hydrazide(3), Pectin Oxadiazole(5) were concerning (σ) absolute softness^59^, (χ) electronegativities^60^, (ΔN_max_) electronic charge^61^, (η) absolute hardness, (ω)^62^ global electrophilicity^63^, (S) global softness^64^, and (Pi) chemical potential^65^, from the equations^1–8^ which were scheduled in Table 4 and Fig. 10A–C^55,66–68^.The analysis of quantum chemistry on Pectin and its derivatives (Pectin Hydrazide and Pectin Oxadiazole) shows that below the surface, the electronics and structure have changed significantly, which governs their chemical reactivity, charge transfer, and overall stability. The total energy (ET) values demonstrate that both derivatives, with pectin hydrazide (-2844.03 a.u.) being the most energetically stable when compared to native pectin (-1650.585 a.u.), due to the enhanced conjugation and introduction of heteroatoms that facilitate delocalized electrons. The frontier orbitals highlight that Pectin Hydrazide has the smallest HOMO–LUMO energy gap (ΔEg = 1.03 eV), and therefore is the most chemically reactive and polarizable. Pectin Oxadiazole has a moderate reactivity with a ΔEg = 5.03 eV and is more stable, and native pectin has the least reactivity with a ΔEg = 6.28 eV. The Hydrazide derivative demonstrates the highest dipole moment (10.04 D) which is consistent with increased polarity and the ability to participate in strong electrostatic or hydrogen-bonding action. It is worth noting that the low relative electronegativity (χ = 1.66 eV) and toughness (η = 0.51 eV) of the derivative compared to pectin is balanced by its comparatively high softness (σ = 1.94 eV⁻^1^) and maximum electron transfer capability (ΔNmax = 7.18). Accordingly, the derivative represents a better (or stronger) Lewis base and Lewis acid than Pectin and thus offers a valuable payload situation for chemistry involving coordination or catalytic interactions^69^. Conversely, Pectin Oxadiazole has a higher electronegativity (χ = 4.70 eV) and hardness (η = 2.52 eV) indicating a more energetically stable and less reactive system, suitable for controlled redox or sensing roles. This rationale is further validated by the electrophilicity index (ω) increasing further from 2.03 eV for pectin to 3.23 eV for Hydrazide, indicating that hydrazide chemistry is suited to charge transfer^54,70,71^. SO, Pectin derivatization creates intermediate Hydrazide and Oxadiazole electronic systems that substantially modify and hone its electronic environment. It will be exciting to see how drew the compositional feature of a chemically inert polysaccharide such as that of Pectin, become derivatives that promote polarity, softness, and redox activity, thereby promoting potential applications for biological binding, metal-coordination, or electrochemical sensing systems.

**Table 4:** The physical descriptors for compounds Pectin (1), Pectin Hydrazide (3), and Pectin Oxadiazole (5) utilizing the DFT/B3LYP/6-31G(d) basis set.

	Pectin	Pectin Hydrazide(3)	Pectin Oxadiazole(5)
E_T_ (au)	– 1650.585	– 2844.03	– 1963.874
E_HOMO_ (eV)	– 6.7114	– 2.17638	– 7.2165
E_LUMO_ (e v)	– 0.4299	– 1.14724	– 2.1842
ΔE_g_ (eV)	6.28152	1.0291	5.0322
µ (D)	6.943	10.043	8.043
χ (eV)	3.57065	1.66181	4.70035
η (eV)	3.14075	0.51457	2.51615
σ (eV)	0.318395	1.94337	0.39743
Pi (eV)	− 3.57065	0.97169	0.19872
S(eV)	0.159198	2.68342	4.3903
ω (eV)	2.02970	3.22951	1.86807
ΔN max	1.13688	7.17762	2.1375

In terms of the Oxadiazole derivative of Pectin, Quantum Atom Theory (QAT) characterizes its dynamism as a delocalized, heteroatom-rich system of electrons residing in quantized molecular orbitals where the spatial probability (Ψ^2^) used for electron occupancy is established in part through the N–O-C heterocycle arrangement and the associated conjugated polysaccharide framework; based on experimentally reported frontier levels (E_HOMO_ = -7.2165 eV, E_LUMO_ = -2.1842 eV) there is a moderate ΔEg = 5.0322 eV which indicates a relatively hard and electronically stable chromophore resists spontaneous charge transfer but admits controlled excitation and redox excitation events; furthermore, because of a repulsion-dipole moment (μ = 8.043D) substantial in part a strongly polar N–O moiety, there is a measure of anisotropic electron density extension toward electrostatically-based (i.e., recognition, directional hydrogen bonding) interactions towards chemical binding at these interface; lastly, while still on conceptual DFT terms, the electronegativity χ = 4.70035 eV, hardness η = 2.51615 eV (softness σ = 0.39743 eV⁻^1^ and global softness S = 4.3903 eV⁻^1^, where provided) do represent its Oxadiazole stability, while also providing a descriptor for somewhat modest polarizability of the molecular acceptor and electronic energy inputs are needed during electron attachment; in particular descriptor of spectroscopic/synthetic interest, where the electrophilicity index ω = 1.86807 eV, speaks to the oxadiazole being distinctly an electrophile that may interact through its n → π* and π / π, but not an over-reactive electrophile^72^. Its chemical potential (Π =  + 0.19872 eV), which is slightly more positive than pectin, suggests an elevated electronic reference point consistent with LUMO character stabilized by heteroatoms, while ΔN_max = 2.1375 suggests that there is a limited ability to transfer charge to a metal, or highly electronegative, reservoir, which can be beneficial for sensing interface, coordination at Lewis acidic sites, or photo/electro induced scenarios where maintaining structural integrity is important, and so on. Overall, the QAT picture reveals an electronically robust, directionally polar heteroatom-conjugated heterocycle, with—HOMO density (dominated by lone-pair/π contributions on heteroatoms, and adjacent π framework) is still deeply bound, LUMO density is lowered and biased toward the Oxadiazole ring making it receptive to charge acceptance, yielding polarity in the medium of high μ, moderate χ, significant η, and modest ω, rendering the Oxadiazole derivative significantly attractive in controlled electron-transfer, recognition, and sensing contexts, where stability and reproducibility are equally important and of equal importance are the affinity terms for bonding^36,73,74^.

When the Pectin Oxadiazole derivative was analyzed using RDG, LOL, ELF, MEP, Fukui and NCI techniques, all are expressed a unique quantum electronic landscape, supporting its profile of reactivity, stability, and intermolecular interactions^75,76^. The Reduced Density Gradient (RDG) mapping indicates a balanced assortment of noncovalent interactions, where areas of deep blue (negative sign λ₂·ρ) suggest a strong hydrogen-bonding and electrostatic attraction around the oxygen and nitrogen atoms of the Oxadiazole moiety, while green surfaces mark the zones of van der Waals dispersion along the conjugated π-ring, revealing the flexibility of the interface for docking. The Localized Orbital Locator (LOL) and Electron Localization Function (ELF) maps displayed the localization of pairs of electrons above the heteroatoms and σ-bonds, with ELF ≈ 0.9 in the lone pairs of som O and N while LOL was lower (0.4–0.6) across the π framework exhibiting delocalized conjugation, critical for charge transfer, with charge transfer being objective of this research.From the Molecular Electrostatic Potential (MEP) perspective, one can infer a large negative potential over the oxygen and nitrogen atoms (red to blue zone)possible nucleophilic sites to undergo coordination or H-bonding and some localized positive portions over hydrogen termini, thereby forming electrophilic sites. This opposite arrangement of charges is responsible for the very high dipole moment (≈ 8 D) and directional binding of the molecule. Fukui function calculations (f⁺, f⁻) further elaborate on this: heteroatoms with large f⁺ values are at the forefront of electrophilic attack, receiving electron density, while the carbonyl carbons with large f⁻ values give nucleophilic character, explaining the amphiphilic reactive nature of the molecule^23,40,77,78^. Finally, the Non-Covalent Interaction (NCI) isosurface plot combines all descriptors basins (attractive H-bonding), green shells (dispersion), and red caps (steric repulsion) to portray how localized and delocalized electron densities compete against each other as they govern binding and self-assembly. The combined quantum studies affirm that Pectin Oxadiazole has a highly polarized and electronically stable yet moderately soft molecular setup with strong directional non with robust directional non-covalent interactions promoting molecular association and biological recognition^2,6,79^. The integration of frontier orbital maps, electrostatic potential surfaces, and localized electron-density analyses illustrates that the presence of the Oxadiazole ring results in pronounced polarization across the entire molecule, resulting in regions where electrophilic and nucleophilic properties are created that enable intermolecular charge transfer. This amphiphilic electronic structure enables simultaneous hydrogen-bond uptake and donation, thus promoting the versatility of interactions with both the hydrophilic and lipophilic portions of a biomolecular target^80–82^. The NCI isosurface also demonstrates balanced pockets of stabilizing (green, van der Waals) and attractive (blue, hydrogen-bonding) interactions paired with localized red steric repulsion, indicating a compact yet flexible electronic framework^36^. Overall, the quantum chemical-derived descriptors and visual analyses demonstrate that Pectin Oxadiazole has a highly polarized, electronically stable, and moderately soft molecular framework capable of directional, multi-site interactions that are ultimately critical to the enhanced reactivity, bio-affinity, and overall superior docking and MD performance of Pectin Oxadiazole over native Pectin and the Pectin Hydrazide analogue^38,83,84^.

## Conclusion

In conclusion, the study has accomplished the synthesis and characterization of novel Pectin Hydrazide and Pectin Oxadiazole derivatives via a two-step reaction of esterification and heterocyclic functionalization of natural Pectin. The structural modification and thermal stability of the derivatives were verified by spectroscopic and thermal properties analyses. The *in vitro *cytotoxicity studies showed both Pectin Oxadiazole and Pectin Hydrazide possess good anti-proliferative activity against the Caco2 colorectal cancer cell line. These derivatives effectively mitigated oxidative stress and angiogenesis by reducing ROS levels and downregulating the NRF2/HO-1, HIF-1α, and VEGF/PDGF-D signaling pathways. Molecular docking and 100-ns molecular dynamics simulations confirmed that the derivatives showed strong and stable binding affinities to several cancer-related targets, especially Pectin Oxadiazole, based on favorable ΔG_bind_ values and small fluctuations in the conformer. In addition, DFT calculations confirmed their enhanced electronic reactivity, justified by the small HOMO–LUMO gap and improved charge transfer ability, consistent with better chemical stability and potential bioactivity. These results collectively demonstrate that heterocyclic derivatization of Pectin yields a multifunctional compound with anticancer, antioxidant, and anti-angiogenic activities with the potential to serve as lead scaffolds for the development of novel therapeutic agents to treat colorectal cancer. In future studies, we aim to evaluate their cytotoxic effects on normal cell lines and perform in vivo toxicity assessments to further confirm their safety profile.

## Supplementary Information

Supplementary Information.

## Data Availability

All data generated or analyzed during this study are included in this published article.
