# Supplementary figures and images for: Multifunctional pectin derivatives as anticancer agents in colorectal cancer via synthesis, computational insights, and modulation of NRF2/HO-1, HIF-1α, and VEGF/PDGF-D signaling pathways
Source: Sci Rep. 2026 Feb 13;16:6542. doi: 10.1038/s41598-025-32107-6 (PMC12910068; doi:10.1038/s41598-025-32107-6)

***Supplementary analysis:***

***1HNMR and 13CNMR of Pectin derivatives:***


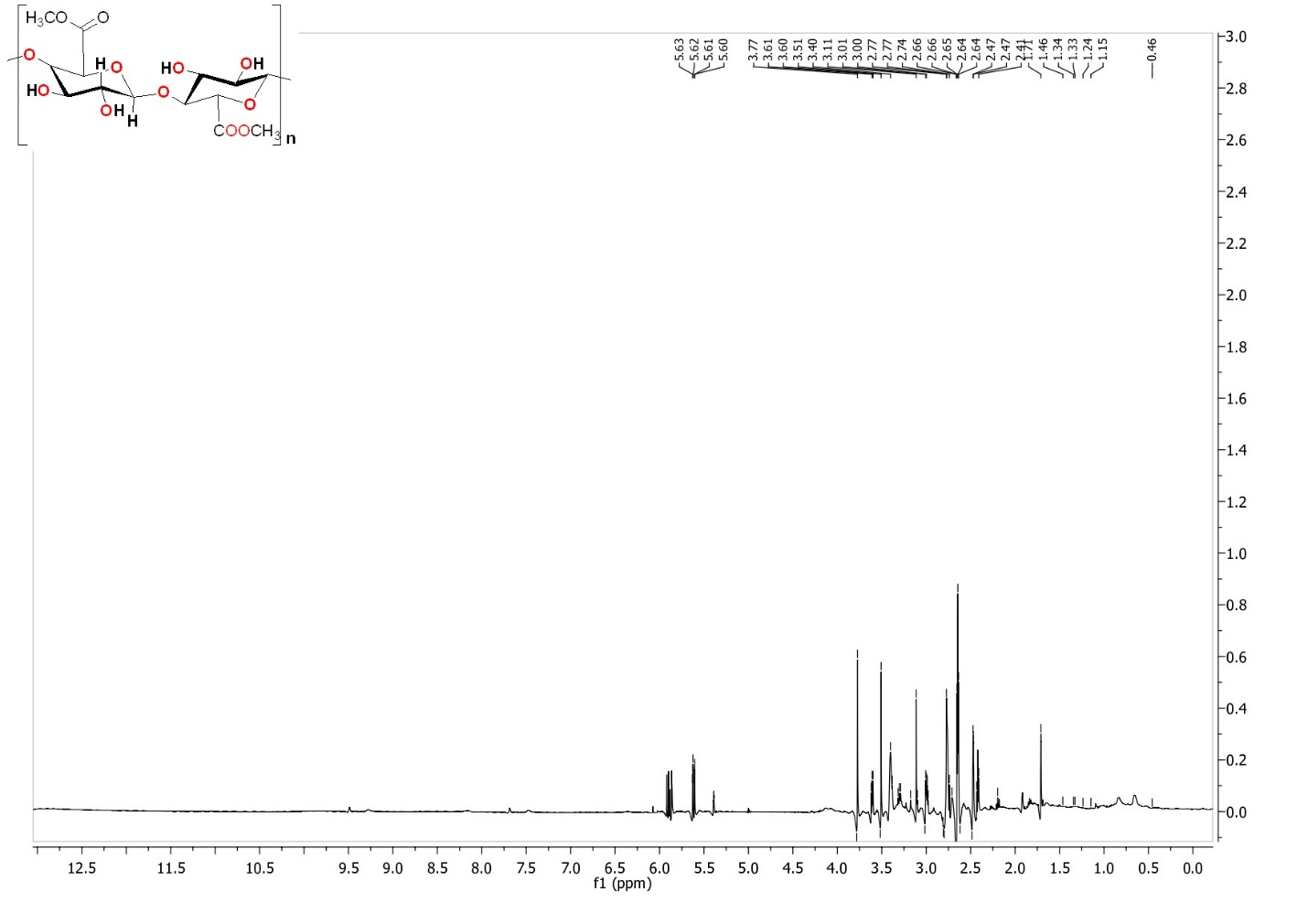


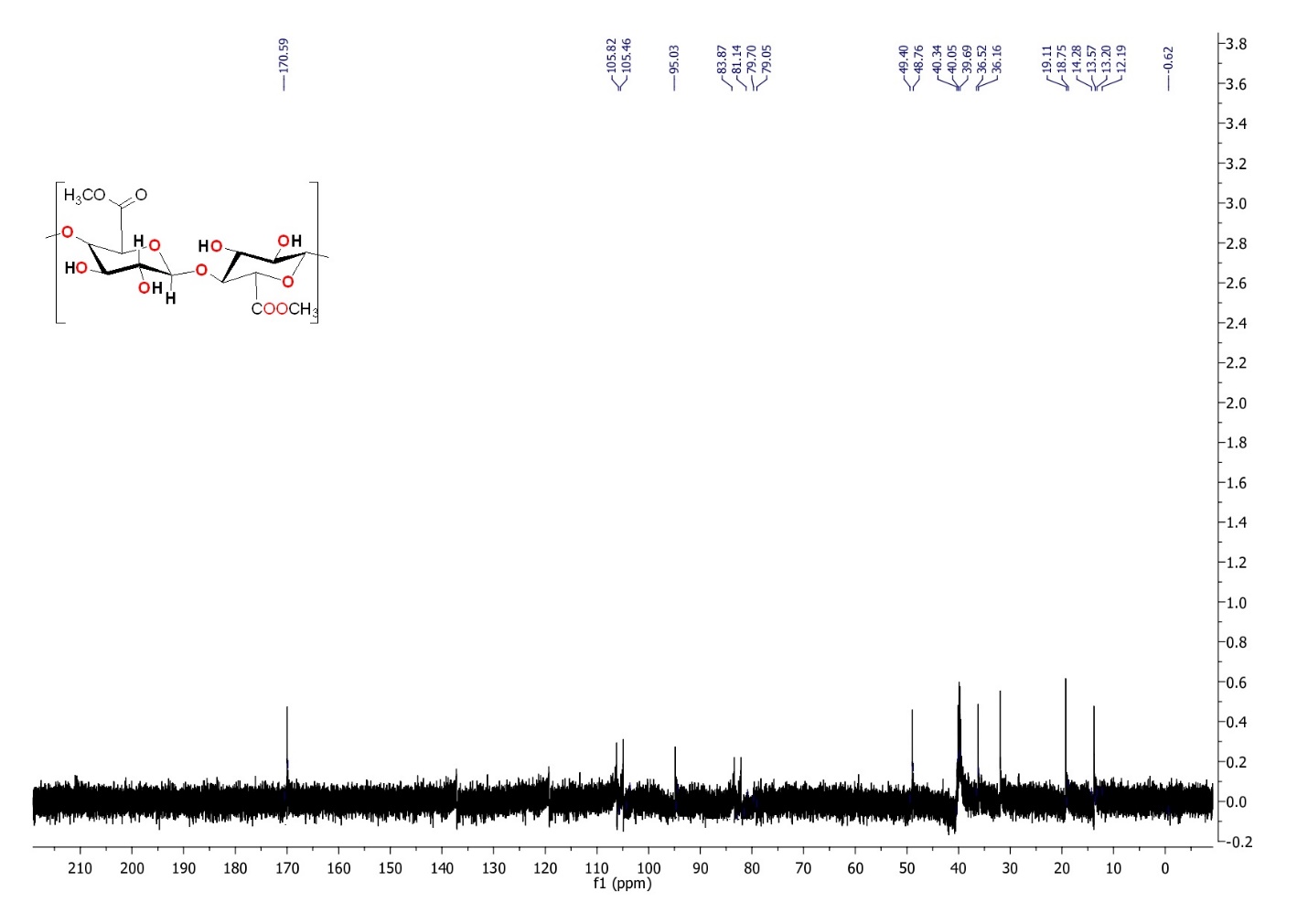


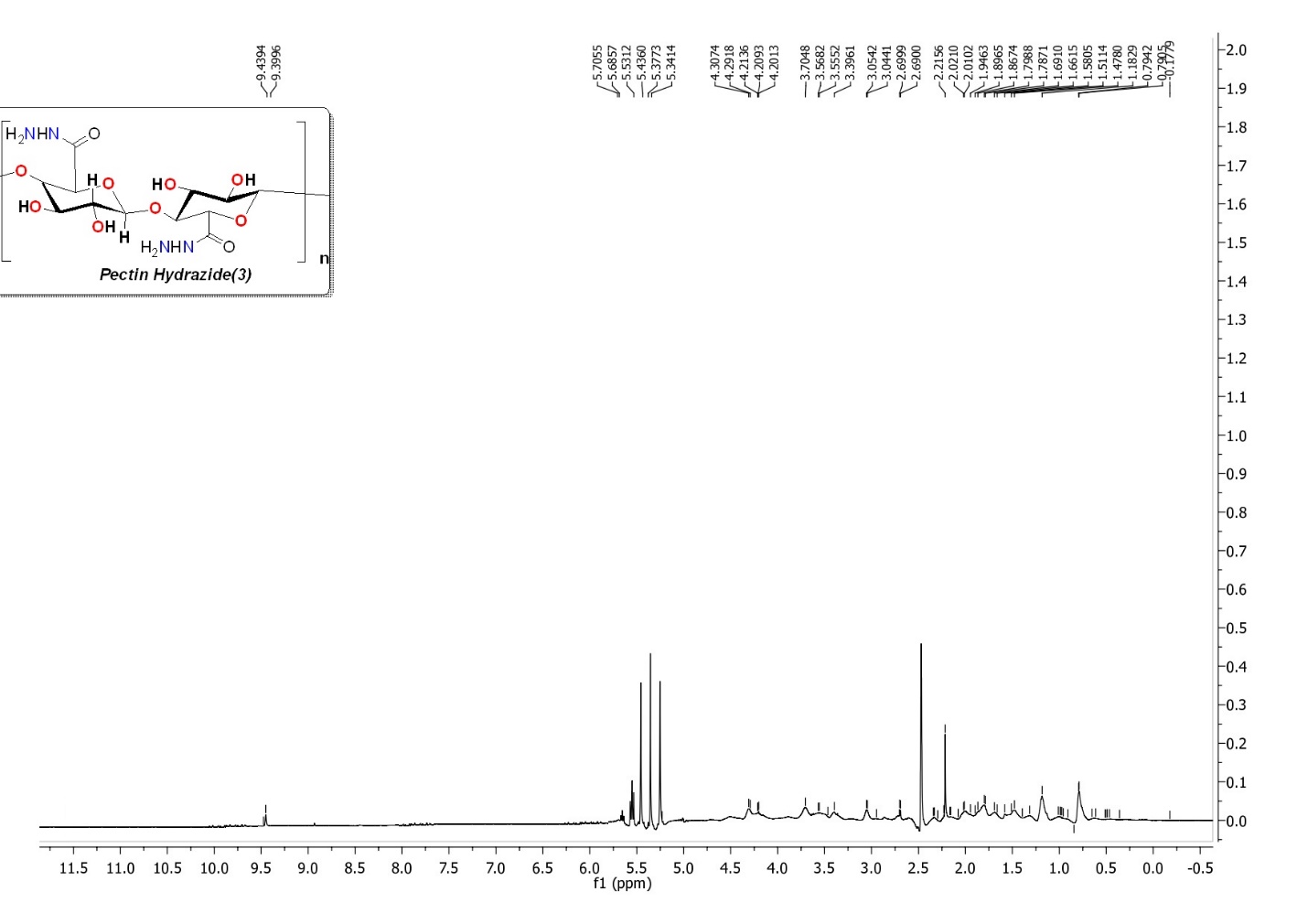


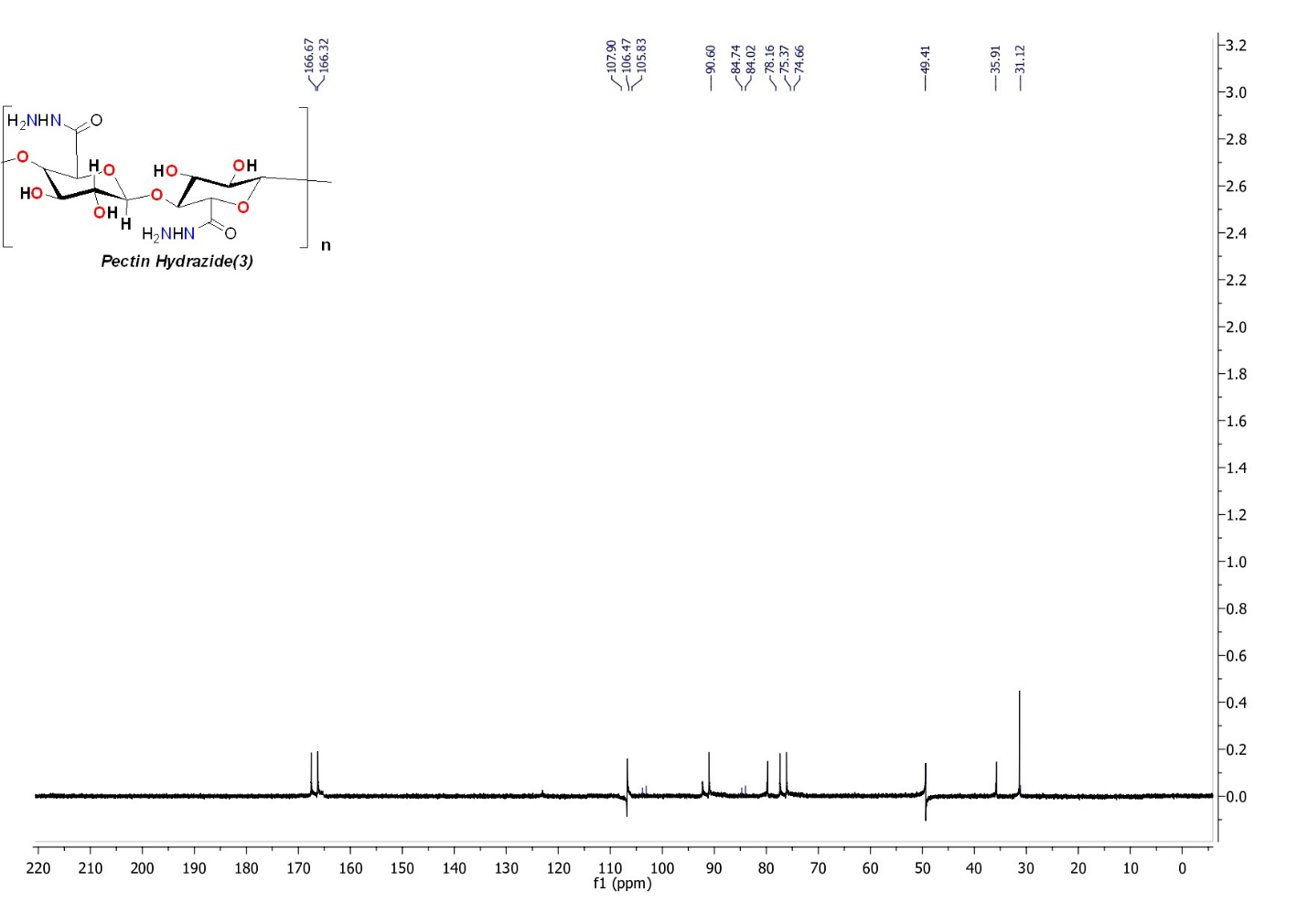


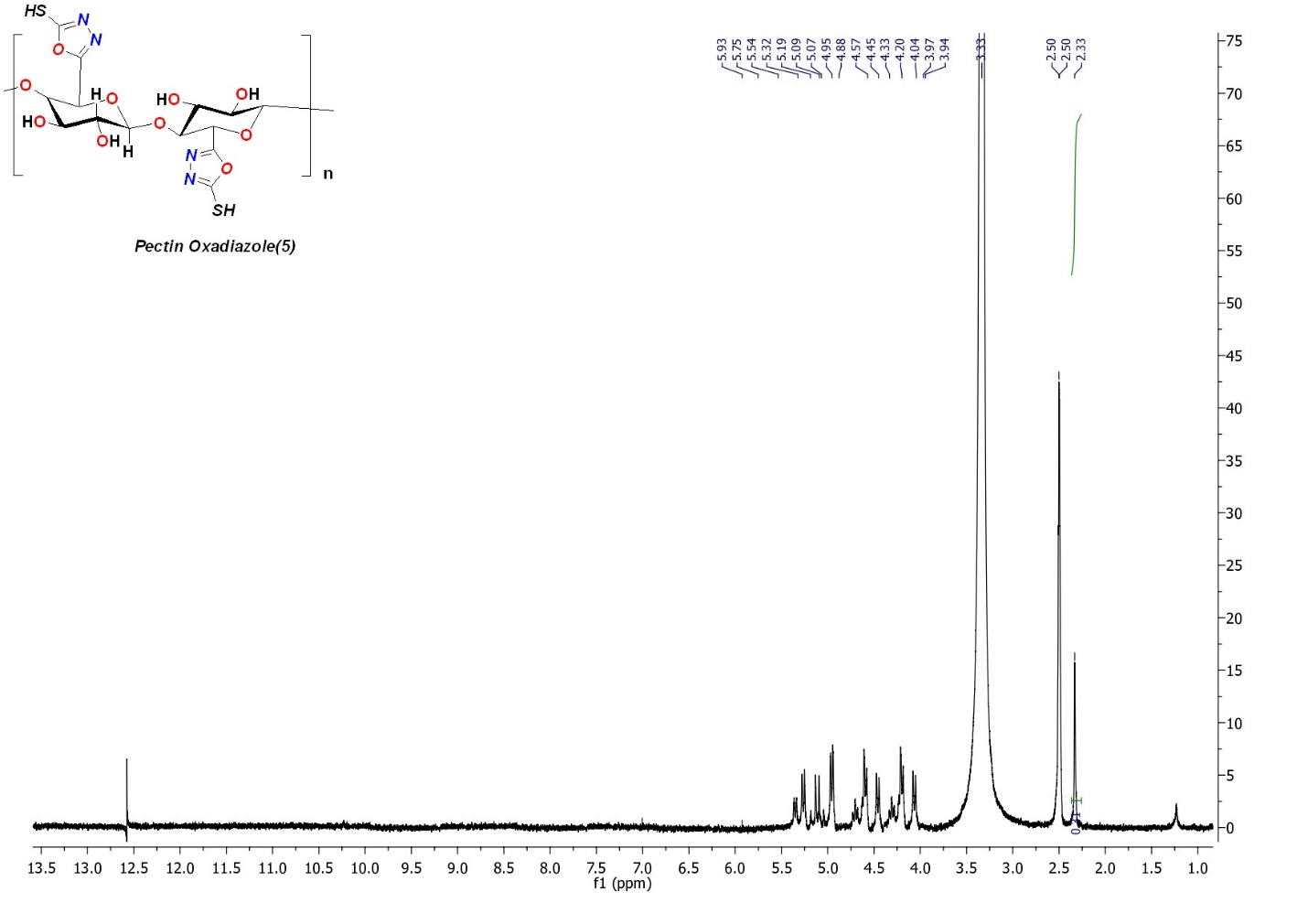


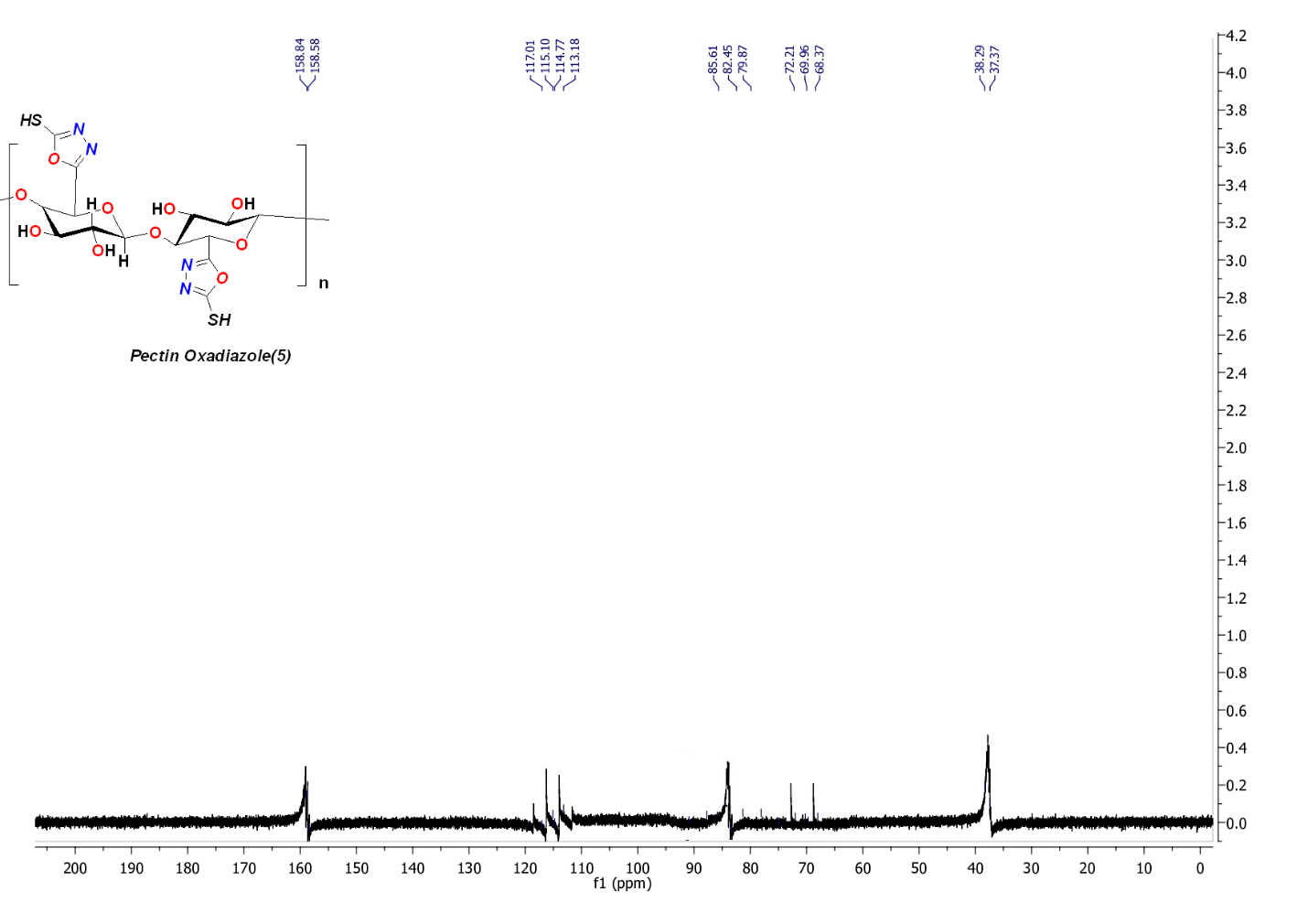

Supplement: Supplementary file 1 — Supplementary Information. [file 41598_2025_32107_MOESM1_ESM.docx]
